# Supplementary material for: Cross-sectional study of prevalence, causes and trends in visual impairment in Nirmal District, Telangana, India: Nirmal Eye Evaluation for Trends study
Source: BMJ Open. 2024 May 30;14(5):e083199. doi: 10.1136/bmjopen-2023-083199 (PMC11141176; doi:10.1136/bmjopen-2023-083199)
Supplement: Supplementary data [file bmjopen-2023-083199supp001.pdf]

ID				

RAPID ASSESSMENT OF VISUAL IMPAIRMENT PROJECT – DATA COLLECTION FORM

Section A – Demographic Informatic

Address:

Name ______ **Age** ______ **Mobile** ______

Status
Circle the codes

1	Examined
2	Not available after 2 visits
3	Refused

Gender

1	Male
2	Female

Education Level

0	No education
1	Primary school (class 1-5)
2	High school (class 6-10)
3	Intermediate (class 11-12)
4	College (undergraduate)
5	Advanced studies (PG etc)
6	Others, specify ______

Occupation

0	Unemployed
1	Teacher / clerical jobs
2	Driver
3	Shop Keeper / Business
4	Labourer – Agriculture
5	Labourer – Other
6	Beedi rolling
7	Stopped working due to poor vision
8	Home duties only
9	Retired / stopped worked due to old age
10	Others, specify ______

Section B – Spectacle Information

Present Glasses

0	No glasses
1	Yes

Amount paid ______

Glasses - Type

0	No glasses
1	SV - Distance
2	SV- Near
3	Bifocals

Provider

0	No Glasses
1	LVP Vision Centre
2	LVP Service centre
3	Private Eye doctor / Clinic
4	Directly from the local optical shop
5	Govt. in a camp for no cost

Section C – Surgery Information and Systemic Conditions (Please enter the codes)

	RE	LE
**When was surgery done**		
**Costs of surgery**		

0	Not applicable
1	Free / Nonpaying
2	Paying **Amount** ______

**Place of surgery**	RE	LE

0	Not applicable	
1	Eye camp	
2	NGO hospital	
3	Private hospital	
4	Government hospital	

Systemic Condition *(Enter the code as appropriate)*

Condition	Duration (yrs)

Codes: 0= None, 1=HTN, 2= DM, 3= Heart disease, 4= Asthma, 5=Other, specify

Marmamula S, *et al. BMJ Open* 2024; 14:e083199. doi: 10.1136/bmjopen-2023-083199

Section D – Visual Acuity and Clinical Examination ***(Enter the code as appropriate)***

**Visual acuity**	**RE**	**LE**	**BE**
Unaided - Distance			
Pinhole - Distance			
(Only if <6/12)			
Aided – Distance*			
Pinhole - Distance			
**PRESENTING VA (PVA)**			

**Lens status**		**RE**	**LE**
1	Normal lens		
2	Obvious lens opacity/cataract		
3	Aphakia		
4	Pseudophakia		
5	No view of lens, why		

Unaided – Near (Binocular)	
Aided - Near	
Add power used	+
Near vision with addition	

* *Record aided vision with +10 for aphakia*

(Codes: cf 3 mts=8; cf 2mts=9; cf 1m=10 PLPR=11; NOPL=12)

**Other Major Finding**			
0	None		
1	Corneal scar		
2	Pterygium		
3	Posterior capsular opacification		
4	Others		

Section E – Primary causes of Visual Impairment ***(Enter the code as appropriate)***

Principal cause of presenting vision <6/12

0	No visual impairment
1	Refractive Error
2	Uncorrected aphakia
3	Cataract
4	Surgery related complications
5	Corneal opacity
6	Phthisis or absent globe
7	Glaucoma
8	Posterior segment disorders
9	Others, specify

**RE**	**LE**	**BIN**

(BIN=least of the two)

Please Note:
Presenting VA= Aided VA if subjects has glasses;
Presenting VA= Unaided VA if subject has no glasses

Name of the examiner: ______________________

Signature/Date: ____________________________

Marmamula S, *et al. BMJ Open* 2024; 14:e083199. doi: 10.1136/bmjopen-2023-083199
