# Supplementary material for: Cross-sectional study of prevalence, causes and trends in visual impairment in Nirmal District, Telangana, India: Nirmal Eye Evaluation for Trends study
Source: BMJ Open. 2024 May 30;14(5):e083199. doi: 10.1136/bmjopen-2023-083199 (PMC11141176; doi:10.1136/bmjopen-2023-083199)
Supplement: Author's manuscript [file bmjopen-2023-083199.draft_revisions.pdf]

BMJ Open

BMJ Open is committed to open peer review. As part of this commitment we make the peer review history of every article we publish publicly available.

When an article is published we post the peer reviewers' comments and the authors' responses online. We also post the versions of the paper that were used during peer review. These are the versions that the peer review comments apply to.

The versions of the paper that follow are the versions that were submitted during the peer review process. They are not the versions of record or the final published versions. They should not be cited or distributed as the published version of this manuscript.

BMJ Open is an open access journal and the full, final, typeset and author-corrected version of record of the manuscript is available on our site with no access controls, subscription charges or pay-per-view fees (http://bmjopen.bmj.com).

If you have any questions on BMJ Open's open peer review process please email info.bmjopen@bmj.com

BMJ Open

Prevalence, causes, and trends in Visual Impairment in Nirmal District, Telangana, India - Nirmal Eye Evaluation for Trends study.

Journal:	*BMJ Open*
Manuscript ID	bmjopen-2023-083199
Article Type:	Original research
Date Submitted by the Author:	14-Dec-2023
Complete List of Authors:	Marmamula, Srinivas; LV Prasad Eye Institute, Gullapalli Pratibha Rao International Centre for Advancement of Rural Eye care; University of New South Wales<br>Chinya, Aritra; LV Prasad Eye Institute<br>Yelagondula, Vijay; LV Prasad Eye Institute, Allen Foster Community Eye Health Research Centre, ICARE<br>Varda, Rajashekar; LV Prasad Eye Institute<br>Khanna, Rohit; LV Prasad Eye Institute<br>Narayanan, Raja; LV Prasad Eye Institute, Vitreo-retina & uveitis
Keywords:	EPIDEMIOLOGIC STUDIES, PUBLIC HEALTH, Observational Study

SCHOLARONE™
Manuscripts

I, the Submitting Author has the right to grant and does grant on behalf of all authors of the Work (as defined in the below author licence), an exclusive licence and/or a non-exclusive licence for contributions from authors who are: i) UK Crown employees; ii) where BMJ has agreed a CC-BY licence shall apply, and/or iii) in accordance with the terms applicable for US Federal Government officers or employees acting as part of their official duties; on a worldwide, perpetual, irrevocable, royalty-free basis to BMJ Publishing Group Ltd ("BMJ") its licensees and where the relevant Journal is co-owned by BMJ to the co-owners of the Journal, to publish the Work in this journal and any other BMJ products and to exploit all rights, as set out in our licence.

The Submitting Author accepts and understands that any supply made under these terms is made by BMJ to the Submitting Author unless you are acting as an employee on behalf of your employer or a postgraduate student of an affiliated institution which is paying any applicable article publishing charge ("APC") for Open Access articles. Where the Submitting Author wishes to make the Work available on an Open Access basis (and intends to pay the relevant APC), the terms of reuse of such Open Access shall be governed by a Creative Commons licence – details of these licences and which Creative Commons licence will apply to this Work are set out in our licence referred to above.

Other than as permitted in any relevant BMJ Author's Self Archiving Policies, I confirm this Work has not been accepted for publication elsewhere, is not being considered for publication elsewhere and does not duplicate material already published. I confirm all authors consent to publication of this Work and authorise the granting of this licence.

For peer review only

ORIGIAL RESEARCH

Prevalence, causes, and trends in Visual Impairment in Nirmal District, Telangana, India - Nirmal Eye Evaluation for Trends study.

Srinivas Marmamula, Ph.D.[1,2,3,4] Aritra Chinya, MPH,[1,2] Vijay Kumar Yelagondula, M.Optom,[2] Rajashekar Varada, MPhil,[1]
Rohit C Khanna, MD[1,4] Raja Narayanan, MS[3,5,6]

[1] Allen Foster Community Eye Health Research Centre, Gullapalli Pratibha Rao International Centre for Advancement of Rural Eye care, L V Prasad Eye Institute, Hyderabad, India

[2] Brien Holden Institute of Optometry and Vision Science, L V Prasad Eye Institute, Hyderabad, India

[3] Wellcome Trust / Department of Biotechnology India Alliance, L V Prasad Eye Institute, Hyderabad, India

[4] School of Optometry and Vision Science, University of New South Wales, Sydney, Australia

[5] Anant Bajaj Retina Institute, L V Prasad Eye Institute, Hyderabad, India

[6] Suven Clinical Research Centre, L V Prasad Eye Institute, Hyderabad, India

Running Title: Visual Impairment in Telangana, India

Keywords: Visual impairment, rapid assessment studies, trends, Universal Eye Health, India

Corresponding author:

Dr. Srinivas Marmamula, Gullapalli Pratibha Rao International Centre for Advancement of Rural Eye care, L V Prasad Eye Institute, Hyderabad, India. 500034

Email: sri.marmamula@lvpei.org

Total number of words:3252 words excluding title page, abstract, acknowledgments, references, tables and figure legends

For peer review only - http://bmjopen.bmj.com/site/about/guidelines.xhtml

ABSTRACT

Background/Aim: To determine the prevalence, causes, and risk factors associated with visual impairment (VI) in the Nirmal district of Telangana, India, using extended Rapid Assessment of Visual Impairment (RAVI) methodology.

Methods: Participants aged ≥16 years were enumerated from 90 randomly selected clusters. Presenting visual acuity (VA) was assessed using a Snellen chart with E optotypes at a six-meter distance was recorded. Near vision was assessed binocularly using an N notation chart with tumbling E optotypes at a 40 cm distance. An anterior segment examination and distance direct ophthalmoscopy were also performed, and non-mydriatic fundus images were obtained. VI was defined as presenting VA worse than 6/12 in the better eye. The prevalence of VI in the current study was compared with a RAVI study conducted in 2014 to assess the trends in VI among those ≥40 years.

Results: In total, 4629/5400 (85.7%) participants were examined. Among them, 55% were women, 53% had at least school-level education, 2.3% self-reported diabetes, and 8.7% self-reported hypertension. The prevalence of VI was 8.81% (95% CI:8.01-9.67). Overall, uncorrected refractive errors (49.5%) were the leading cause of VI, followed by cataracts (40.2%) and posterior segment diseases (4.9%). Among those aged ≥40 years, the prevalence of VI declined by 19.3% compared to the 2014 baseline study (20.2% to 16.3%; $p<0.01$).

Conclusion: The extended RAVI study conducted in the Nirmal district showed a considerable decline in the prevalence of VI. Targeted interventions are needed to provide adequate eye care for the high-risk groups in this district.

For peer review only

ARTICLE SUMMARY:

Strengths and limitations of the study

- Visual impairment is a public health challenge affecting a large proportion of people in the Indian state of Telangana.
- Rapid assessments typically focus on participants aged ≥40 years. This study extends the rapid assessment methodology to include your age groups (≥40 years) and provides estimates on the prevalence and causes of visual impairment.
- In addition to prevalence estimates, temporal trends in the prevalence of visual impairment are presented.
- The overrepresentation of women could have overestimated the prevalence of VI in our study.

For peer review only

For peer review only - http://bmjopen.bmj.com/site/about/guidelines.xhtml

INTRODUCTION

Over a billion people suffer from needless vision impairment (VI) globally, with cataracts and uncorrected refractive errors (URE) being the leading causes.[1,2] However, these conditions can be addressed using cost-effective interventions, such as spectacles and cataract surgery. Population-based data on the prevalence and causes of VI are essential to plan eye care service models to address this global problem. Though conventional epidemiological studies provide the data, they are often resource-intensive and need expertise to implement them. The rapid assessment methods are low-cost epidemiological tools that provide data on the prevalence and causes of VI using limited resources while being relatively easy to implement. In addition, these rapid assessments can be repeated at stipulated intervals to study the temporal trends in a given region.[3] Rapid assessment studies are even more important now, with WHO setting global targets for effective cataract surgical coverage and effective refractive error coverage as indicators to measure the progress toward Universal Eye Health.[4]

Rapid assessment studies initially focused on cataract alone; however, they were modified and evolved to cover other causes of VI, with an increasing focus on emerging eye conditions, such as diabetic retinopathy and refractive errors.[3,5] Rapid Assessment of Visual Impairment (RAVI) is an offshoot of multiple rapid assessment methods developed for eye care, and has been used extensively in India and other countries.[6-11] Studies using the RAVI methodology focus on individuals aged 40 years and older. Recently, it has been modified to include younger individuals (≥16 years) and has been renamed as the extended RAVI methodology.[12] In addition, new tools have been added to collect data on systemic conditions and disabilities, helping to more holistic planning of holistic eye health programs.[5,13] The Nirmal Eye Evaluation for Trends is the first study to use extended RAVI. In this study, we report the prevalence, causes, and risk factors of VI in the Nirmal district and adjoining areas of Telangana, India. In addition to VI, this paper also compares the temporal trend in the prevalence of VI in this region using data from a previous study conducted in 2014.

MATERIALS AND METHODS

Ethics Approval

The study protocol was approved by the Institutional Review Board (IRB) of Hyderabad Eye Research Foundation, L V Prasad Eye Institute (LVPEI) (Reference ID:LEC-08173). This

For peer review only - http://bmjopen.bmj.com/site/about/guidelines.xhtml

study was conducted in accordance with the tenets of the Declaration of Helsinki. Written informed consent was obtained from all the participants (or the legal guardian as applicable) before enrolment in the study.

Patient/participant involvement

The study participants were not involved in setting the research question or the outcome measures.

Sampling strategy

Assuming a VI prevalence of 3.5% (presenting visual acuity worse than 6/12), allowing for a 95% confidence interval, a precision of 20%, a design effect of 1.6 for a predetermined cluster size of 60 participants, and a 20% non-response rate, the minimum sample size required was 5270, which was rounded up to 5400 (90 clusters) participants. A multi-stage cluster random sampling procedure with a compact segment sampling method was used, which has been described in previous reports.[14] The study area had a population of 0.5 million people and comprised ten sub-districts (mandal) from the Nirmal (eight) and Nizamabad (two) districts. The eye care needs of the study area were serviced by a secondary centre of LVPEI. Data were collected between November 2021 and March 2022.

Data collection

Three teams, comprising a vision technician and two community eye health workers, collected the data. They were supervised by a study coordinator (optometrist), who was also responsible for travel logistics and quality control. The examiners were trained to conduct the study procedures and document the findings. A reliability assessment was conducted before the study to assess the inter-observer agreement on visual acuity with a gold-standard senior optometrist. All examiners had a good agreement with the gold-standard optometrist (kappa 0.8 or more).

One of the three study teams visited participants at their homes and conducted eye examinations. The time of the visits were planned to maximize the availability of the participants at their households for examination. In each selected household, all the individuals who fulfilled the age criteria were documented, and all those who were available during the visit were examined. At least two attempts were made to examine those who were unavailable during the first visit, after which they were marked as unavailable.

For peer review only - http://bmjopen.bmj.com/site/about/guidelines.xhtml

Eye Examination protocol

A brief interview was conducted to collect personal and social-demographic information, such as age, education level, and systemic health conditions. Data related to the ocular history, including current and previous use of spectacles, use of eye drops, and details of any previous surgery, were also collected. Information regarding the barriers to the uptake of eye care services was also collected using a structured questionnaire.

The standard RAVI clinical examination was conducted after the interview, as described in previous studies.[11,15-17] In brief, the distance visual acuity (VA) was assessed using a standard Snellen chart with tumbling E optotypes at a distance of six meters. If a participant was unable to identify the letters in the first line of the chart, the distance between the participant and the chart was progressively reduced to three meters and then one meter till VA could be recorded. Unaided VA was recorded for all participants. Aided VA was recorded for participants using spectacles for correction. Aided VA was considered as the presenting VA for those with spectacles, and unaided VA was considered as presenting VA. If the presenting VA was worse than 6/12, the VA was recorded using a multiple pinhole occluder. Near vision was assessed binocularly using the N notation chart at a fixed distance of 40 cm in ambient lighting conditions. The fixed distance was maintained using a string attached to the near vision chart. Both unaided and aided near vision were assessed if the participant reported spectacles use. Near vision was re-assessed using near addition lenses in a trial frame appropriate for that age among participants with near vision worse than N8.

An external eye examination was performed using a torchlight/portable slit lamp. The lens was assessed using distant direct ophthalmoscopy in a shaded area (indoors), which was graded as normal, obvious lens opacity, aphakia, pseudophakia without posterior capsular opacification (PCO), or pseudophakia with PCO. If the lens could not be examined because of corneal opacities, phthisis bulbi, or absent globe, then it was documented in the data form. A non-mydriatic portable fundus camera (Visuscout 100 Handheld Fundus Camera, Carl Zeiss Meditec, USA) was used to capture retinal images. Two images, one optic disc-centred and another macula-centred, were captured for each eye. All the images were evaluated by experienced graders at L V Prasad Eye Institute. The participants with VI and those requiring other eye care services were referred to the nearest eye care facility for management.

For peer review only - http://bmjopen.bmj.com/site/about/guidelines.xhtml

The WHO categorizes visual impairment (VI) into four categories based on the presenting visual acuity in the better eye.[18] The four categories are as follows: Mild VI (MiVI - VA worse than 6/12 to 6/18), Moderate VI (MVI - VA worse than 6/18 to 6/60), Severe VI (SVI - worse than 6/60 to 3/60), and Blindness (VA worse than 3/60 to no perception of light). The case definitions for the causes of VI used in this study have been described in our previous publications.[11] In brief, uncorrected refractive error was defined as presenting VA <6/12, improving to 6/12 or better with pinhole. Cataract was defined as an opacity of the crystalline lens as seen with torchlight and obscuring the red reflex, partially or completely, on the distance direct ophthalmoscopy, resulting in a VA <6/12 that does not improve with pinhole. Posterior segment disease was considered as the cause of VI in cases where there was no media opacity and visual acuity did not improve with a pinhole. Posterior capsular opacification, corneal opacities/edema after cataract surgery were marked as surgical complications. After the eye examination, the principal cause of VI was recorded for each eye separately, and then for the person. If there was more than one cause, the cause that was more easily treatable or correctable was marked as the main cause of visual impairment.

As this study was conducted during the pandemic, all COVID-19-related protocols were followed, including the use of masks (N-95) and visors at all times, frequent hand sanitization, and social distancing. All the team members were vaccinated before the start of the study. The equipment used, such as trial frames and multiple pinhole occluders, were disinfected with alcohol wipes/swabs after each use. The participants were offered hand sanitizer to clean their hands before starting the study procedures. The current health status of all the participants was enquired before the eye examinations.

For peer review only

Data Management

In the field, data were collected using paper forms. The forms were then transported to the data centre for entry into a Microsoft Access database. Data analyses were performed using the Stata Statistical Software for Windows, version 14 (StataCorp, College Station, TX). The prevalence estimates were adjusted to the age and gender distribution of the population for the year 2011, which have been presented with the 95% confidence intervals (CI). The demographic associations of VI with age, gender, education, and systemic conditions were assessed using multiple logistic regression models and adjusted odds ratios (OR) with 95% CI. A study using the conventional RAVI methodology was conducted in the same region in

For peer review only - http://bmjopen.bmj.com/site/about/guidelines.xhtml

2014.[14] The prevalence estimates from the current study were compared with the 2014 study to assess the trends in VI over time in this region.

RESULTS

Characteristics of the participants.

Of the 5400 participants included in this study from the 90 clusters, 4629 (85.7%) were examined. The mean age (±standard deviation) of the examined participants was similar to those not examined (42.5 (±16.6) years versus 42.0 (±16.5) years; p=0.38). A higher proportion of women were examined (55% versus 44%; p<0.01). Among those examined, 55% (n=2545) were women, 53% (2456) had at least school education, 2.3% (n=129) self-reported diabetes, and 8.7% (n=402) self-reported hypertension (Table 1).

Table 1: Visual impairment and demographic characteristics of the participants

	Total examined (n)	Number of participants with VI n (%)	p-value
**Age group (years)**			<0.01
16-29	1,244	12 (1.0)	
30-39	1,010	12 (1.2)	
40-49	899	30 (3.3)	
50-59	684	81 (11.8)	
60-69	440	127 (28.9)	
70 & above	352	146 (41.5)	
**Gender**			0.11
Male	2084	199 (9.5)	
Female	2545	209 (8.2)	
**Education level**			<0.01
No education	2173	356 (16.4)	
Any education	2456	52 (2.1)	
**Diabetes**			<0.01
Yes	129	35 (27.1)	
No	4500	373 (8.3)	
**Hypertension**			<0.01
Yes	402	73 (18.2)	
No	4227	335 (7.9)	
**Total**	**4629**	**408 (8.8)**	

The overall crude prevalence of VI was 8.81% (95% CI:8.01 – 9.67). This included MiVI (2.87%; 95% CI:2.41 – 3.40), MVI (4.6%; 95% CI:4.06 – 5.29), SVI (0.60%; 95% CI:0.40 –

For peer review only - http://bmjopen.bmj.com/site/about/guidelines.xhtml

0.87), and blindness (0.69%; 95% CI: 0.47 – 0.97). Age and gender adjusted prevalence was 7.15% (95% CI: 6.80 – 8.32) (Table 2).

Table 2: Crude prevalence and age and gender adjusted prevalence of visual impairment (VI).

	**Crude prevalence (95% Confidence Intervals)**	**Age and gender adjusted prevalence (95% Confidence Intervals)**
Mild VI	2.87 (2.41 - 3.40)	2.37 (2.00 - 2.90)
Moderate VI	4.64 (4.06 - 5.29)	4.03 (3.50 - 4.64)
Severe VI	0.60 (0.40 - 0.87)	0.55 (0.35 - 0.80)
Blind	0.69 (0.47 - 0.97)	0.57 (0.37 - 0.82)
**All VI**	**8.81 (8.01 - 9.67)**	**7.51 (6.80 - 8.32)**

Risk factors for VI

On univariate analysis, the prevalence of VI was highest (41.2%) in the oldest age group. Though the prevalence of VI did not vary with gender (p=0.11), it was significantly higher among those with no formal education (16.4% versus 2.1%; p<0.01). The prevalence of VI was also higher among those who self-reported hypertension (18.2% versus 7.9%; p<0.01) and diabetes (27.1% versus 8.3%; p<0.01).

The multiple regression analysis showed that the odds for VI increased with increasing age. Compared to the participants aged 16-29 years, the odds for VI were 6.78 (95% CI: 3.46–13.29) for the 50-59 age group, 6.7 (95% CI: 3.4–13.2) for the 60-69 age group, and 33.7 (95% CI: 17.19–66.37) in the above 70 and older age group. The participants with no formal education had higher odds for VI compared to those with formal education (OR: 2.75; 95% CI: 1.90–3.97). Similarly, the participants with a history of diabetes had higher odds for VI (OR: 1.83; 95% CI: 1.16–2.89). Women had lower odds for VI compared to men (OR: 0.64; 95% CI: 0.51–0.82). Hypertension was not associated with VI (p=0.321) (Table 3).

For peer review only - http://bmjopen.bmj.com/site/about/guidelines.xhtml

Table 3: Effects of socio-demographic variables in visual impairment (multiple logistic regression analysis)

	Adjusted Odds Ratio (95% Confidence Intervals)	p-value
**Age group (years)**		
16- 29	Reference	
30-39	0.93 (0.4 - 2.11)	0.865
40-49	2.02 (0.99 - 4.13)	0.053
50-59	6.78 (3.46 -13.29)	<0.01
60-69	18.83 (9.6 -36.87)	<0.01
>=70	33.77 (17.19 - 66.37)	<0.01
**Gender**		
Male	Reference	
Female	0.64 (0.51- 0.82)	<0.01
**Education**		
Any education	Reference	
No education	2.75 (1.90 – 3.97)	<0.01
**Hypertension**		
No	Reference	
Yes	0.85 (0.62 - 1.17)	0.321
**Diabetes**		
No	Reference	
Yes	1.83 (1.16 - 2.89)	0.01

Causes of VI

Overall, uncorrected refractive errors (49.5%) were the leading cause of VI, followed by cataracts (40.2%) and posterior segment diseases (4.9%). Uncorrected refractive errors were the leading cause of moderate and severe VI, and cataract were the leading cause of blindness. Uncorrected refractive errors were the leading cause of VI in the younger age group (16-59 years), and cataract were the leading cause of VI in the older age group (60 years and older) (Figure 1).

Temporal trends in VI.

The data of individuals aged ≥40 years examined in the 2014 and 2021-2022 studies were analyzed to capture the trends in the prevalence of VI over time. In total, 2,974 and 2,375 participants aged ≥40 years were examined in the 2014 and 2021-2022 studies, respectively. The mean age (±standard deviation) of the participants was higher in the 2022 study (55.0 ±11.0 years versus 51.7±9.9 years; p<0.05). Similarly, lesser proportion of men were examined in the 2022 study (42.5% versus 45.6%; p=0.03). Overall, the prevalence of VI declined by 19.3% compared to the 2014 baseline study (20.2% to 16.3%; p<0.01). In terms

For peer review only - http://bmjopen.bmj.com/site/about/guidelines.xhtml

of categories of VI, MiVI declined by 21.9% (from 6.4% to 5.0%; p=0.03), MSVI declined by 15.1% (from 11.9% to 10.1%; p=0.04), and blindness declined by 36.8% (from 1.9% to 1.6%).

DISCUSSION

We have reported on the prevalence and causes of VI among the adult population in the Nirmal district of Telangana using the extended RAVI methodology. The conventional RAAB and RAVI methods include individuals aged ≥50 years and ≥40 years, respectively. In contrast, the extended RAVI methodology used in this study included anyone ≥16 years. While it is advantageous to only include the older population to minimize the sample size and use of resources, the data on VI is not readily available in the younger age groups. Data on all ages is essential to plan universal eye health initiatives in the region. The extended RAVI is an attempt to provide comprehensive information on the prevalence of VI in the complete adult population in this region. The data from this study can supplement the data from school eye health programs, providing a complete picture of the entire population, other than children under five years. In addition, we used the revised WHO definitions in this study for cross-comparison with other studies done in India and other regions of the world.

The Andhra Pradesh Eye Disease Study (APEDS) conducted between 1996 and 2000 was the only population-based cross-sectional study that included the population of all ages. The prevalence of Moderate VI, Severe VI, and blindness were 10.1%, 2.3%, and 2.3%, respectively. [19,20] Using similar definitions, the prevalence of Moderate VI, Severe VI, and blindness in this study were 4.6%, 0.60%, and 0.69%, respectively.[19,21] The prevalence of Mild VI is not reported in APEDS. Despite a difference in the age groups between the studies, a lower prevalence in this study indicates a decline in the prevalence of VI in this region over the last three decades. Such a secular trend of decline in VI has been reported from various locations, suggesting an improvement in the availability and uptake of eye care services in this region.

Both APEDS and the current study had a higher prevalence of VI among the older participants, which is consistent across all the studies conducted in this region.[19,21] In this study, though the prevalence did not vary with gender, women had lower odds for VI, which is contrary to the APEDS study, where women had a higher prevalence of VI. This difference could be attributed to availability, acceptability, and a higher uptake of eye care services

For peer review only - http://bmjopen.bmj.com/site/about/guidelines.xhtml

among women. A higher prevalence of VI was also noted among those with lower levels of education, which is similar to other studies in this region.[11,14,19,21] The higher visual needs and availability of resources for eye examinations and treatment might have attributed to a lower prevalence of VI among those with higher levels of education. The participants who self-reported diabetes had a higher prevalence of VI in this study, which might be caused by the earlier incidence of cataract secondary to diabetes and other refractive changes in the eye.

Several studies have reported the prevalence of VI using the RAVI methodology among participants aged ≥40 years in Andhra Pradesh, Telangana, and other parts of India.[14,15, 22,23,17-24] The prevalence of VI ranges from 8.7% in Tripura,[24] 10% in Krishna district in Andhra Pradesh,[15] 11.4% in Delhi,[22] 12.8% in Ganjam and Khordha districts in Odisha,[23] 12.8% in Akividu region West Godavari and Krishna districts,[17] and 13.7% in Mahbubnagar and Adilabad districts in Telangana.[14] These are comparable to the 11.3% found in the current study.

Uncorrected refractive errors and cataract remain the leading causes of VI.[11,14,17, 24-26] Similarly to APEDS, uncorrected refractive errors are the leading causes of VI in the older age groups compared to the younger age groups.[19,21] Moreover, similar to other studies, cataract was more common among those with severe grades of VI.[11,14,17, 25,26] As both these conditions are manageable with cost-effective interventions, strategies are needed to reach these communities and provide eye care.

The temporal trends on the prevalence of VI have been reported from Telangana.[26-28] In an earlier paper, we compared two studies conducted using the same methodology and geographical locations, Khammam and Warangal districts, that included identical age groups.[26] There was a 2.5% decline in VI in Khammam district, but it remained stable in Warangal district over a five-year period.[26] In this study, we observed an 19% decline in VI compared to the study conducted in 2014, which is an annual decline of 2%. This decline could be attributed to increased availability and uptake of eye care services in this region. However, due to the absence of a control arm, the role of secular trends resulting in the decline of VI cannot be ruled out.

For peer review only - http://bmjopen.bmj.com/site/about/guidelines.xhtml

The Nirmal district (erstwhile Adilabad district) has witnessed a few epidemiological studies over the years. The Andhra Pradesh Eye Disease Study (APEDS) was conducted in 1997-98, followed by Rapid Assessment of Cataract Surgical Services (RACSS), a couple of RAVI studies in 2014, and the current study in 2022 (Table 4). Among those aged ≥40 years, the prevalence of VI (<6/18 definition) was 35.2% in APEDS, which dropped to 13.7% in the 2014 RAVI study. The prevalence declined further to 9.7% in the current study. Among those aged 50 years and older, the prevalence of VI (<6/18 definition) was 50.7% in APEDS, which dropped to 21.6% in RACSS conducted in 2006-07, and remained stable in the 2014 RAVI study, declining to 16.2% in the current study. As different protocols have been used over the years, direct comparisons are limited by the different measurement methods. Nevertheless, VI is declining in this region, as indicated by the two recent RAVI studies using identical protocols.

Table 4: Prevalence of visual impairment in Nirmal district by reported various studies.

	**Year**	**Sample size**	**Moderate Visual Impairment** (Presenting visual acuity worse than 6/18 to 6/60)	**Severe Visual Impairment** (Presenting visual acuity worse than 6/60 to 3/60)	**Blindness** (Presenting visual acuity worse than 3/60)	**Total Visual Impairment** (Presenting visual acuity worse than 6/18)
		**n**	**%**	**%**	**%**	**%**
**≥40 years**						
APEDS*	1997-98	840	28.5	1.8	5.0	35.2
RAVI †	2014	2974	10.4	1.5	1.9	13.7
RAVI †	2022	2392	8.8	0.4	0.6	9.7
**≥50 years**						
APEDS *	1997-98	521	40.3	2.9	7.5	50.7
RACSS ∫	2006-07	2160	13.6	4.8	3.2	21.6
RAVI †	2014	1550	16.6	2.3	3.0	21.9
RAVI †	2022	1491	13.4	1.1	1.7	16.2

** Andhra Pradesh Eye Disease Study. †Rapid Assessment of Visual Impairment. ∫ Rapid Assessment of Cataract Surgical Services.*

A good response rate, a randomly selected population-based sample, the use of an updated WHO definition of VI, and the inclusion of participants of wider age groups are the strengths of this study. After APEDS, this is the major study to report the VI in the adult population in this region. However, a few studies have reported the VI in older age groups during this gap

For peer review only - http://bmjopen.bmj.com/site/about/guidelines.xhtml

of two decades. A higher proportion of women were examined in this study, which could be due to the migration of men to urban areas in search of work, a common occurrence in Telangana. The previous studies in this region also show a female preponderance.[11,14] Therefore, the overrepresentation of women could have overestimated the prevalence of VI in our study.

In conclusion, a significant burden of VI is observed in the region. However, the declining trend in the prevalence of VI suggests that the eye care services in the region are improving. This study can be a guide for more focused efforts to address vision loss and achieve universal eye health in this region. Also, there is need for integration and eye care services with primary health care as people with diabetes had a higher prevalence of VI. VI impedes the attainment of sustainable development goals and the overall quality of life. Efforts to address vision loss might have a ripple effect on the overall health and well-being of individuals, families, and communities, contributing towards sustainable development goals.

Acknowledgments: The authors thank the vision technicians (Sunil Anant Doke, Vaibhav Kumar Dave, Jalindar Kumar Doke, Degaon Ganesh, Gangadharolla Vamshi) and community eye health team (Yedla Sandeep, Akaram Chinnaiah, Laxman Sunkari, Gunde Rao Rahul, Ramnapoina Mahesh) for the assistance provided in data collection. The authors also thank Sandeep Dayal, Thirupathi Reddy Kumbham, Shekhar Konegari and Saptak Banerjee for monitoring the study. Abhinav Sekar is acknowledged for the language inputs on earlier versions of our manuscript. The authors thank the volunteers for their participation in the study.

Contributors: SM conceived the idea, designed and conducted the study, analyzed the data and wrote the manuscript. AC was involved in data collection and quality control. VKY, RSV, RCK, RN reviewed the earlier version of the manuscripts and provided the intellectual inputs.

Funding Support: This work was supported by the DBT Wellcome Trust India Alliance Clinical Research Centre Grant [grant number IA/CRC/19/1/610010] awarded to Dr Raja Narayanan and Hyderabad Eye Research Foundation, India.

Competing Interests: The authors report no conflicts of interest and have no proprietary interest in any of the materials mentioned in this article.

For peer review only - http://bmjopen.bmj.com/site/about/guidelines.xhtml

REFERENCES

1. Bourne R, Steinmetz JD, Flaxman S, et al. Trends in prevalence of blindness and distance and near vision impairment over 30 years: an analysis for the Global Burden of Disease Study. The Lancet Global Health 2021;**9**(2):e130-e43 doi: https://doi.org/10.1016/S2214-109X(20)30425-3[published Online First: Epub Date]|.
2. Steinmetz JD, Bourne RRA, Briant PS, et al. Causes of blindness and vision impairment in 2020 and trends over 30 years, and prevalence of avoidable blindness in relation to VISION 2020: the Right to Sight: an analysis for the Global Burden of Disease Study. The Lancet Global Health 2021;**9**(2):e144-e60 doi: 10.1016/S2214-109X(20)30489-7[published Online First: Epub Date]|.
3. Marmamula S, Keeffe JE, Rao GN. Rapid assessment methods in eye care: An overview. Indian J Ophthalmol 2012;**60**(5):416-22 doi: IndianJOphthalmol_2012_60_5_416_100539 [pii]
10.4103/0301-4738.100539[published Online First: Epub Date]|.
4. McCormick I, Mactaggart I, Resnikoff S, et al. Eye health indicators for universal health coverage: results of a global expert prioritisation process. Br J Ophthalmol 2022;**106**(7):893-901 doi: 10.1136/bjophthalmol-2020-318481[published Online First: Epub Date]|.
5. Mactaggart I, Limburg H, Bastawrous A, Burton MJ, Kuper H. Rapid Assessment of Avoidable Blindness: looking back, looking forward. Br J Ophthalmol. 2019;**103**:1549-52
6. Marmamula S, Khanna RC, Narsaiah S, Shekhar K, Rao GN. Prevalence of spectacle use in Andhra Pradesh, India: Rapid Assessment of Visual Impairment (RAVI) project. Clin Exp Ophthalmol 2013 doi: 10.1111/ceo.12160[published Online First: Epub Date]|.
7. Marmamula S, Madala SR, Rao GN. Rapid assessment of visual impairment (RAVI) in marine fishing communities in South India--study protocol and main findings. BMC Ophthalmol 2011;**11**:26 doi: 10.1186/1471-2415-11-26[published Online First: Epub Date]|.
8. Marmamula S, Madala SR, Rao GN. Prevalence of uncorrected refractive errors, presbyopia and spectacle coverage in marine fishing communities in South India: Rapid Assessment of Visual Impairment (RAVI) project. Ophthalmic Physiol Opt 2012;**32**(2):149-55 doi: 10.1111/j.1475-1313.2012.00893.x[published Online First: Epub Date]|.

For peer review only - http://bmjopen.bmj.com/site/about/guidelines.xhtml

9. Marmamula S, Narsaiah S, Shekhar K, Khanna RC. Visual Impairment among Weaving Communities in Prakasam District in South India. PLoS One 2013;**8**(2):e55924 doi: 10.1371/journal.pone.0055924[published Online First: Epub Date]|.

10. Marmamula S, Narsaiah S, Shekhar K, Khanna RC. Presbyopia, spectacles use and spectacle correction coverage for near vision among cloth weaving communities in Prakasam district in South India. Ophthalmic Physiol Opt 2013 doi: 10.1111/opo.12079[published Online First: Epub Date]|.

11. Marmamula S, Narsaiah S, Shekhar K, Khanna RC, Rao GN. Visual Impairment in the South Indian State of Andhra Pradesh: Andhra Pradesh - Rapid Assessment of Visual Impairment (AP-RAVI) Project. PLoS One 2013;**8**(7):e70120 doi: 10.1371/journal.pone.0070120[published Online First: Epub Date]|.

12. Marmamula S, Yelagondula VK. The feasibility of combining rapid assessment studies for measuring the progress toward Universal Eye Health. Indian J Ophthalmol 2023;**71**(5):2296-97 doi: 10.4103/IJO.IJO_2996_22[published Online First: Epub Date]|.

13. Marmamula S, Modepalli SB, Kumbham TR, Challa R, Keeffe JE. Prevalence of disabilities and non-communicable diseases in an elderly population in the Telangana state, India: a population-based cross-sectional study. BMJ open 2021;**11**(2):e041755 doi: 10.1136/bmjopen-2020-041755[published Online First: Epub Date]|.

14. Marmamula S, Khanna RC, Kunkunu E, Rao GN. Population-based assessment of prevalence and causes of visual impairment in the state of Telangana, India: a cross-sectional study using the Rapid Assessment of Visual Impairment (RAVI) methodology. BMJ open 2016;**6**(12):e012617 doi: 10.1136/bmjopen-2016-012617[published Online First: Epub Date]|.

15. Marmamula S, Khanna RC, Narsaiah S, Shekhar K, Rao GN. Prevalence of spectacles use in Andhra Pradesh, India: rapid assessment of visual impairment project. Clin Exp Ophthalmol 2014;**42**(3):227-34 doi: 10.1111/ceo.12160[published Online First: Epub Date]|.

16. Marmamula S, Khanna RC, Shekhar K, Rao GN. A population-based cross-sectional study of barriers to uptake of eye care services in South India: the Rapid Assessment of Visual Impairment (RAVI) project. BMJ open 2014;**4**(6):e005125 doi: 10.1136/bmjopen-2014-005125[published Online First: Epub Date]|.

17. Marmamula S, Yelagondula VK, Khanna RC, et al. A Population-Based Cross-Sectional Study of Visual Impairment in West Godavari and Krishna Districts in Andhra

For peer review only

Pradesh: Akividu Visual Impairment Study (AVIS). Ophthalmic Epidemiol 2022;**29**(4):411-16 doi: 10.1080/09286586.2021.1946829[published Online First: Epub Date]|.

18. Flaxman SR, Bourne RRA, Resnikoff S, et al. Global causes of blindness and distance vision impairment 1990-2020: a systematic review and meta-analysis. Lancet Glob Health 2017;**5**(12):e1221-e34 doi: 10.1016/S2214-109X(17)30393-5[published Online First: Epub Date]|.
19. Dandona L, Dandona R, Srinivas M, et al. Blindness in the Indian state of Andhra Pradesh. Invest Ophthalmol Vis Sci 2001;**42**(5):908-16
20. Dandona R, Dandona L, Srinivas M, et al. Moderate visual impairment in India: the Andhra Pradesh Eye Disease Study. Br J Ophthalmol 2002;**86**(4):373-7
21. Dandona L, Dandona R, Naduvilath TJ, et al. Burden of moderate visual impairment in an urban population in southern India. Ophthalmology 1999;**106**(3):497-504
22. Gupta N, Vashist P, Malhotra S, Senjam SS, Misra V, Bhardwaj A. Rapid assessment of visual impairment in urban population of Delhi, India. PLoS One 2015;**10**(4):e0124206 doi: 10.1371/journal.pone.0124206[published Online First: Epub Date]|.
23. Bhardwaj A, Vashist P, Singh Senjam S, Gupta V, Gupta N, Manna S. Rapid Assessment of Avoidable Visual Impairment in Two Coastal Districts of Eastern India for Determining Effective Coverage: A Cross-Sectional Study. J Ophthalmic Vis Res 2023;**18**(2):182-91 doi: 10.18502/jovr.v18i2.13185[published Online First: Epub Date]|.
24. Marmamula S, Yellapragada S, Khanna RC. Population Based Assessment of Prevalence and Causes of Vision Impairment in the North-eastern State of Tripura, India - The Tripura Eye Survey. Ophthalmic Epidemiol 2020;**27**(4):283-88 doi: 10.1080/09286586.2020.1731833[published Online First: Epub Date]|.
25. Senjam SS, Vashist P, Gupta N, et al. Prevalence of visual impairment due to uncorrected refractive error: Results from Delhi-Rapid Assessment of Visual Impairment Study. Indian J Ophthalmol 2016;**64**(5):387-90 doi: 10.4103/0301-4738.185614[published Online First: Epub Date]|.
26. Marmamula S, Khanna RC, Yellapragada S, Challa R, Mohd J, Rao GN. Temporal trends in the prevalence and causes of visual impairment in the South Indian state of Telangana: a population-based cross-sectional study. BMJ open 2019;**9**(7):e029114 doi: 10.1136/bmjopen-2019-029114[published Online First: Epub Date]|.

For peer review only - http://bmjopen.bmj.com/site/about/guidelines.xhtml

For peer review only

27. Khanna RC, Marmamula S, Krishnaiah S, Giridhar P, Chakrabarti S, Rao GN. Changing trends in the prevalence of blindness and visual impairment in a rural district of India: Systematic observations over a decade. Indian J Ophthalmol 2012;**60**(5):492-7 doi: IndianJOphthalmol_2012_60_5_492_100560 [pii]
10.4103/0301-4738.100560[published Online First: Epub Date]|.

28. Marmamula S, Keeffe JE, Narsaiah S, Khanna RC, Rao GN. Changing trends in the prevalence of visual impairment, uncorrected refractive errors and use of spectacles in Mahbubnagar district in South India. Indian J Ophthalmol 2013;**61**(12):755-8 doi: 10.4103/0301-4738.121149[published Online First: Epub Date]|.

Figure 1: Causes of visual impairment across the age groups.

For peer review only

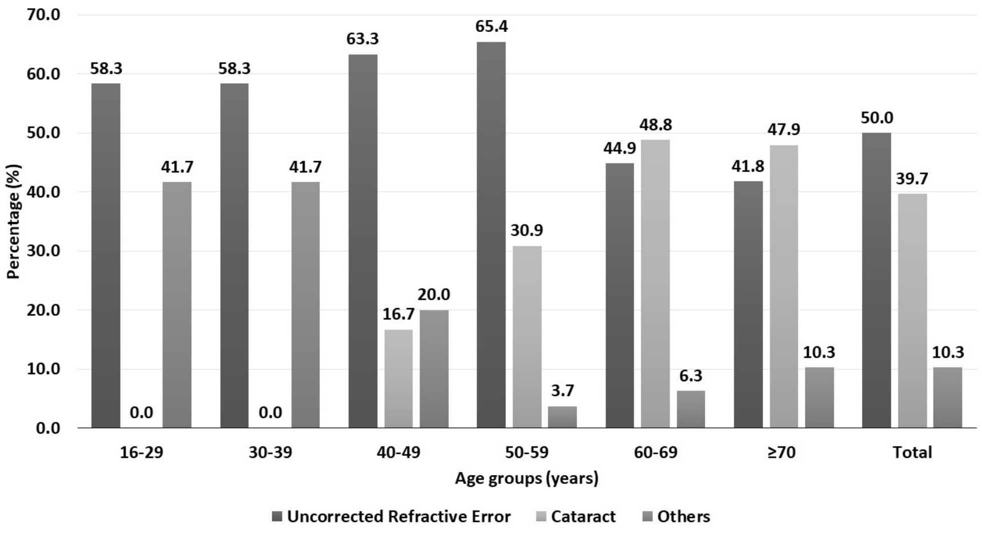

Causes of visual impairment across the age groups

338x190mm (96 x 96 DPI)

STROBE 2007 (v4) Statement—Checklist of items that should be included in reports of *cross-sectional studies*

Section/Topic	Item #	Recommendation	Reported on page #
**Title and abstract**	1	(*a*) Indicate the study's design with a commonly used term in the title or the abstract	1
		(*b*) Provide in the abstract an informative and balanced summary of what was done and what was found	1
**Introduction**			
Background/rationale	2	Explain the scientific background and rationale for the investigation being reported	3
Objectives	3	State specific objectives, including any pre-specified hypotheses	3
**Methods**			
Study design	4	Present key elements of study design early in the paper	3
Setting	5	Describe the setting, locations, and relevant dates, including periods of recruitment, exposure, follow-up, and data collection	4
Participants	6	(*a*) Give the eligibility criteria, and the sources and methods of selection of participants	4
Variables	7	Clearly define all outcomes, exposures, predictors, potential confounders, and effect modifiers. Give diagnostic criteria, if applicable	4,5
Data sources/ measurement	8*	For each variable of interest, give sources of data and details of methods of assessment (measurement). Describe comparability of assessment methods if there is more than one group	4,5
Bias	9	Describe any efforts to address potential sources of bias	NA
Study size	10	Explain how the study size was arrived at	na
Quantitative variables	11	Explain how quantitative variables were handled in the analyses. If applicable, describe which groupings were chosen and why	6
Statistical methods	12	(*a*) Describe all statistical methods, including those used to control for confounding	6
		(*b*) Describe any methods used to examine subgroups and interactions	6
		(*c*) Explain how missing data were addressed	NA
		(*d*) If applicable, describe analytical methods taking account of sampling strategy	NA
		(*e*) Describe any sensitivity analyses	NA
**Results**			

For peer review only

Participants	13*	(a) Report numbers of individuals at each stage of study—eg numbers potentially eligible, examined for eligibility, confirmed eligible, included in the study, completing follow-up, and analysed	NA
		(b) Give reasons for non-participation at each stage	NA
		(c) Consider use of a flow diagram	NA
Descriptive data	14*	(a) Give characteristics of study participants (eg demographic, clinical, social) and information on exposures and potential confounders	7
		(b) Indicate number of participants with missing data for each variable of interest	NA
Outcome data	15*	Report numbers of outcome events or summary measures	7
Main results	16	(*a*) Give unadjusted estimates and, if applicable, confounder-adjusted estimates and their precision (eg, 95% confidence interval). Make clear which confounders were adjusted for and why they were included	7,8,9
		(*b*) Report category boundaries when continuous variables were categorized	6
		(*c*) If relevant, consider translating estimates of relative risk into absolute risk for a meaningful time period	6
Other analyses	17	Report other analyses done—eg analyses of subgroups and interactions, and sensitivity analyses	7,8,9
**Discussion**			
Key results	18	Summarise key results with reference to study objectives	10
Limitations	19	Discuss limitations of the study, taking into account sources of potential bias or imprecision. Discuss both direction and magnitude of any potential bias	12,13
Interpretation	20	Give a cautious overall interpretation of results considering objectives, limitations, multiplicity of analyses, results from similar studies, and other relevant evidence	12,13
Generalisability	21	Discuss the generalisability (external validity) of the study results	13
**Other information**			
Funding	22	Give the source of funding and the role of the funders for the present study and, if applicable, for the original study on which the present article is based	13

*Give information separately for cases and controls in case-control studies and, if applicable, for exposed and unexposed groups in cohort and cross-sectional studies.

Note: An Explanation and Elaboration article discusses each checklist item and gives methodological background and published examples of transparent reporting. The STROBE checklist is best used in conjunction with this article (freely available on the Web sites of PLoS Medicine at http://www.plosmedicine.org/, Annals of Internal Medicine at http://www.annals.org/, and Epidemiology at http://www.epidem.com/). Information on the STROBE Initiative is available at www.strobe-statement.org.

BMJ Open

A cross-sectional study of prevalence, causes, and trends in visual impairment in Nirmal District, Telangana, India - Nirmal Eye Evaluation for Trends study.

Journal:	*BMJ Open*
Manuscript ID	bmjopen-2023-083199.R1
Article Type:	Original research
Date Submitted by the Author:	06-Mar-2024
Complete List of Authors:	Marmamula, Srinivas; LV Prasad Eye Institute, Gullapalli Pratibha Rao International Centre for Advancement of Rural Eye care; University of New South Wales<br>Chinya, Aritra; LV Prasad Eye Institute<br>Yelagondula, Vijay; LV Prasad Eye Institute, Allen Foster Community Eye Health Research Centre, ICARE<br>Varda, Rajashekar; LV Prasad Eye Institute<br>Khanna, Rohit; LV Prasad Eye Institute<br>Narayanan, Raja; LV Prasad Eye Institute, Vitreo-retina & uveitis
**Primary Subject Heading**:	Ophthalmology
Secondary Subject Heading:	Epidemiology, Public health
Keywords:	EPIDEMIOLOGIC STUDIES, PUBLIC HEALTH, Observational Study

SCHOLARONE™ Manuscripts

I, the Submitting Author has the right to grant and does grant on behalf of all authors of the Work (as defined in the below author licence), an exclusive licence and/or a non-exclusive licence for contributions from authors who are: i) UK Crown employees; ii) where BMJ has agreed a CC-BY licence shall apply, and/or iii) in accordance with the terms applicable for US Federal Government officers or employees acting as part of their official duties; on a worldwide, perpetual, irrevocable, royalty-free basis to BMJ Publishing Group Ltd ("BMJ") its licensees and where the relevant Journal is co-owned by BMJ to the co-owners of the Journal, to publish the Work in this journal and any other BMJ products and to exploit all rights, as set out in our licence.

The Submitting Author accepts and understands that any supply made under these terms is made by BMJ to the Submitting Author unless you are acting as an employee on behalf of your employer or a postgraduate student of an affiliated institution which is paying any applicable article publishing charge ("APC") for Open Access articles. Where the Submitting Author wishes to make the Work available on an Open Access basis (and intends to pay the relevant APC), the terms of reuse of such Open Access shall be governed by a Creative Commons licence – details of these licences and which Creative Commons licence will apply to this Work are set out in our licence referred to above.

Other than as permitted in any relevant BMJ Author's Self Archiving Policies, I confirm this Work has not been accepted for publication elsewhere, is not being considered for publication elsewhere and does not duplicate material already published. I confirm all authors consent to publication of this Work and authorise the granting of this licence.

For peer review only

ORIGINAL RESEARCH

A cross-sectional study of prevalence, causes, and trends in visual impairment in Nirmal District, Telangana, India - Nirmal Eye Evaluation for Trends study.

Srinivas Marmamula, Ph.D.[1,2,3,4] Aritra Chinya, MPH,[1,2] Vijay Kumar Yelagondula, M.Optom,[2] Rajashekar Varada, MPhil,[1]
Rohit C Khanna, MD[1,4] Raja Narayanan, MS[3,5,6]

[1] Allen Foster Community Eye Health Research Centre, Gullapalli Pratibha Rao International Centre for Advancement of Rural Eye care, L V Prasad Eye Institute, Hyderabad, India

[2] Brien Holden Institute of Optometry and Vision Science, L V Prasad Eye Institute, Hyderabad, India

[3] Wellcome Trust / Department of Biotechnology India Alliance, L V Prasad Eye Institute, Hyderabad, India

[4] School of Optometry and Vision Science, University of New South Wales, Sydney, Australia

[5] Anant Bajaj Retina Institute, L V Prasad Eye Institute, Hyderabad, India

[6] Suven Clinical Research Centre, L V Prasad Eye Institute, Hyderabad, India

Running Title: Visual Impairment in Telangana, India

Keywords: Visual impairment, rapid assessment studies, trends, Universal Eye Health, India

Corresponding author:

Dr. Srinivas Marmamula, Gullapalli Pratibha Rao International Centre for Advancement of Rural Eye care, L V Prasad Eye Institute, Hyderabad, India. 500034

Email: sri.marmamula@lvpei.org

Total number of words:3252 words excluding title page, abstract, acknowledgements, references, tables and figure legends

For peer review only - http://bmjopen.bmj.com/site/about/guidelines.xhtml

ABSTRACT

Objective: To determine the prevalence, causes, and risk factors associated with visual impairment (VI) in the Nirmal district of Telangana, India, using extended Rapid Assessment of Visual Impairment (RAVI) methodology.

Design: Cross-sectional study

Setting: Community setting

Participants: Participants aged ≥16 years were enumerated from 90 randomly selected clusters and 4629/5400 (85.7%) participants were examined. Presenting visual acuity (VA) was assessed using a Snellen chart with E optotypes at a six-meter distance was recorded. Near vision was assessed binocularly using an N notation chart with tumbling E optotypes at a 40 cm distance. An anterior segment examination and distance direct ophthalmoscopy at 50 cm were also performed, and non-mydriatic fundus images were obtained. VI was defined as presenting VA worse than 6/12 in the better eye. The prevalence of VI in the current study was compared with a RAVI study conducted in 2014 to assess the trends in VI among those ≥40 years.

Primary outcome: Prevalence, causes and risk factors for VI.

Results: Among those examined, 55% were women, 53% had at least school-level education, 2.3% self-reported diabetes, and 8.7% self-reported hypertension. The prevalence of VI was 8.81% (95% CI:8.01-9.67). Overall, uncorrected refractive errors (49.5%) were the leading cause of VI, followed by cataracts (40.2%) and posterior segment diseases (4.9%). Among those aged ≥40 years, the prevalence of VI declined by 19.3% compared to the 2014 baseline study (20.2% to 16.3%; $p<0.01$).

Conclusion: The extended RAVI study conducted in the Nirmal district showed a considerable decline in the prevalence of VI. Targeted interventions are needed to provide adequate eye care for the high-risk groups in this district.

For peer review only

For peer review only - http://bmjopen.bmj.com/site/about/guidelines.xhtml

ARTICLE SUMMARY:

Strengths and limitations of the study

- Rapid assessments typically focus on participants aged ≥40 years. This study extends the rapid assessment methodology to include younger age groups (≥40 years) and provides estimates on the prevalence and causes of visual impairment.
- In addition to prevalence estimates, temporal trends in the prevalence of visual impairment are presented**.**
- As a randomly selected population-based sample was used, the results from the study can be extrapolated to the population in the region.
- The overrepresentation of women could have overestimated the prevalence of VI in our study.

For peer review only

INTRODUCTION

Over a billion people suffer from needless vision impairment (VI) globally, with cataracts and uncorrected refractive errors (URE) being the leading causes.[1,2] However, these conditions can be addressed using cost-effective interventions, such as spectacles and cataract surgery. Population-based data on the prevalence and causes of VI are essential to plan eye care service models to address this global problem. Though conventional epidemiological studies provide the data, they are often resource-intensive and need expertise to implement them. The rapid assessment methods are low-cost epidemiological tools that provide data on the prevalence and causes of VI using limited resources while being relatively easy to implement. In addition, these rapid assessments can be repeated at stipulated intervals to study the temporal trends in a given region.[3] Rapid assessment studies are even more important now, with WHO setting global targets for effective cataract surgical coverage and effective refractive error coverage as indicators to measure the progress toward Universal Eye Health.[4]

Rapid assessment studies initially focused on cataract alone; however, they were modified and evolved to cover other causes of VI, with an increasing focus on emerging eye conditions, such as diabetic retinopathy and refractive errors.[3,5] Rapid Assessment of Visual Impairment (RAVI) is an offshoot of multiple rapid assessment methods developed for eye care and has been used extensively in India and other countries.[6-11] STUDIES using the RAVI methodology focus on individuals aged 40 years and older. Recently, it has been modified to include younger individuals (≥16 years) and has been renamed as the extended RAVI methodology.[12] In addition, new tools have been added to collect data on systemic conditions and disabilities, helping to more holistic planning of holistic eye health programs.[5,13] The Nirmal Eye Evaluation for Trends is the first study to use extended RAVI. In this study, we report the prevalence, causes, and risk factors of VI in the Nirmal district and adjoining areas of Telangana, India. In addition to VI, this paper also compares the temporal trend in the prevalence of VI in this region using data from a previous study conducted in 2014.[14]

MATERIALS AND METHODS

Ethics Approval

The study protocol was approved by the Institutional Review Board (IRB) of Hyderabad Eye Research Foundation, L V Prasad Eye Institute (LVPEI) (Reference ID: LEC-08173). This

For peer review only - http://bmjopen.bmj.com/site/about/guidelines.xhtml

study was conducted in accordance with the tenets of the Declaration of Helsinki. Written informed consent was obtained from all the participants (or the legal guardian if the age of the participant is less than 18 years) before enrolment in the study.

Patient and public involvement

Patients and other members of the public were not involved in the design of the study.

Sampling strategy

Assuming a VI prevalence of 3.5% (presenting visual acuity worse than 6/12), allowing for a 95% confidence interval, a precision of 20%, a design effect of 1.6 for a predetermined cluster size of 60 participants, and a 20% non-response rate, the minimum sample size required was 5270, which was rounded up to 5400 (90 clusters) participants. A multi-stage cluster random sampling procedure with a compact segment sampling method was used, which has been described in previous reports.[14] The study area had a population of 0.5 million people and comprised ten sub-districts (mandal) from the Nirmal (eight) and Nizamabad (two) districts. The eye care needs of the study area were serviced by a secondary centre of LVPEI. Data were collected between November 2021 and March 2022.

Data collection

Three teams, comprising a vision technician and two community eye health workers, collected the data. They were supervised by a study coordinator (optometrist), who was also responsible for travel logistics and quality control. The examiners were trained to conduct the study procedures and document the findings. A reliability assessment was conducted before the study to assess the inter-observer agreement on visual acuity with a gold-standard senior optometrist. All examiners had a good agreement with the gold-standard optometrist (kappa 0.8 or more).

One of the three study teams visited participants at their homes and conducted eye examinations. The time of the visits was planned to maximize the availability of the participants at their households for examination. In each selected household, all the individuals who fulfilled the age criteria were documented, and all those who were available during the visit were examined. At least two attempts were made to examine those who were unavailable during the first visit, after which they were marked as unavailable.

For peer review only - http://bmjopen.bmj.com/site/about/guidelines.xhtml

Eye Examination protocol

A brief interview was conducted to collect personal and social-demographic information, such as age, education level, and systemic health conditions. (supplemental file - 1) Data related to the ocular history, including current and previous use of spectacles, use of eye drops, and details of any previous surgery, were also collected. Information regarding the barriers to the uptake of eye care services was also collected using a structured questionnaire.

The standard RAVI clinical examination was conducted after the interview, as described in previous studies.[11,15-17] In brief, the distance visual acuity (VA) was assessed using a standard Snellen chart with tumbling E optotypes at a distance of six meters. If a participant was unable to identify the letters in the first line of the chart, the distance between the participant and the chart was progressively reduced to three meters and then one meter till VA could be recorded. Unaided VA was recorded for all participants. Aided VA was recorded for participants using spectacles for correction. Aided VA was considered as the presenting VA for those with spectacles, and unaided VA was considered as the presenting VA. If the presenting VA was worse than 6/12, the VA was recorded using a multiple pinhole occluder. Near vision was assessed binocularly using the N notation chart at a fixed distance of 40 cm in ambient lighting conditions. The fixed distance was maintained using a string attached to the near vision chart. Both unaided and aided near vision were assessed if the participant reported spectacles use. Near vision was re-assessed using near addition lenses in a trial frame appropriate for that age among participants with near vision worse than N8.

An external eye examination was performed using a torchlight/portable slit lamp. The lens was assessed using distant direct ophthalmoscopy at about 50 cm distance in a shaded area (indoors), which was graded as normal, obvious lens opacity, aphakia, pseudophakia without posterior capsular opacification (PCO), or pseudophakia with PCO. If the lens could not be examined because of corneal opacities, phthisis bulbi, or absent globe, then it was documented in the data form. A non-mydriatic portable fundus camera (Visuscout 100 Handheld Fundus Camera, Carl Zeiss Meditec, USA) was used to capture retinal images. Two images, one optic disc-centred and another macula-centred, were captured for each eye. All the images were evaluated by experienced graders at L V Prasad Eye Institute. The participants with VI and those requiring other eye care services were referred to the nearest eye care facility for management.

For peer review only - http://bmjopen.bmj.com/site/about/guidelines.xhtml

The WHO categorizes visual impairment (VI) into four categories based on the presenting visual acuity in the better eye.[18] The four categories are as follows: Mild VI (MiVI - VA worse than 6/12 to 6/18), Moderate VI (MVI - VA worse than 6/18 to 6/60), Severe VI (SVI - worse than 6/60 to 3/60), and Blindness (VA worse than 3/60 to no perception of light). The case definitions for the causes of VI used in this study have been described in our previous publications.[11] In brief, uncorrected refractive error was defined as presenting VA <6/12, improving to 6/12 or better with pinhole. Cataract was defined as an opacity of the crystalline lens as seen with torchlight and obscuring the red reflex, partially or completely, on the distance direct ophthalmoscopy, resulting in a VA <6/12 that does not improve with pinhole. Posterior segment disease was considered as the cause of VI in cases where there was no media opacity and visual acuity did not improve with a pinhole. Posterior capsular opacification, and corneal opacities/edema after cataract surgery were marked as surgical complications. After the eye examination, the principal cause of VI was recorded for each eye separately, and then for the person. If there was more than one cause, the cause that was more easily treatable or correctable was marked as the main cause of visual impairment.

As this study was conducted during the pandemic, all COVID-19-related protocols were followed, including the use of masks (N-95) and visors at all times, frequent hand sanitization, and social distancing. All the team members were vaccinated before the start of the study. The equipment used, such as trial frames and multiple pinhole occluders, were disinfected with alcohol wipes/swabs after each use. The participants were offered hand sanitiser to clean their hands before starting the study procedures. The current health status of all the participants was enquired before the eye examinations.

Data Management

In the field, data were collected using paper forms. The forms were then transported to the data centre for entry into a Microsoft Access database. Data analyses were performed using the Stata Statistical Software for Windows, version 14 (StataCorp, College Station, TX). The prevalence estimates were adjusted to the age and gender distribution of the population for the year 2011, which have been presented with the 95% confidence intervals (CI). The demographic associations of VI with age, gender, education, and systemic conditions were assessed using multiple logistic regression models and adjusted odds ratios (OR) with 95% CI. A study using the conventional RAVI methodology was conducted in the same region in

For peer review only - http://bmjopen.bmj.com/site/about/guidelines.xhtml

2014.[14] The prevalence estimates from the current study were compared with the 2014 study to assess the trends in VI over time in this region.

RESULTS

Characteristics of the participants.

Of the 5400 participants included in this study from the 90 clusters, 4629 (85.7%) were examined. The mean age (±standard deviation) of the examined participants was similar to those not examined (42.5 (±16.6) years versus 42.0 (±16.5) years; p=0.38). A higher proportion of women were examined (55% versus 44%; p<0.01). Among those examined, 55% (n=2545) were women, 53% (2456) had at least school education, 2.3% (n=129) self-reported diabetes, and 8.7% (n=402) self-reported hypertension (Table 1).

Table 1: Visual impairment and demographic characteristics of the participants

	Total examined (n)	Number of participants with VI n (%)	p-value
**Age group (years)**			<0.01
16-29	1,244	12 (1.0)	
30-39	1,010	12 (1.2)	
40-49	899	30 (3.3)	
50-59	684	81 (11.8)	
60-69	440	127 (28.9)	
70 & above	352	146 (41.5)	
**Gender**			0.11
Male	2084	199 (9.5)	
Female	2545	209 (8.2)	
**Education level**			<0.01
No education	2173	356 (16.4)	
Any education	2456	52 (2.1)	
**Diabetes**			<0.01
Yes	129	35 (27.1)	
No	4500	373 (8.3)	
**Hypertension**			<0.01
Yes	402	73 (18.2)	
No	4227	335 (7.9)	
**Total**	**4629**	**408 (8.8)**	

The overall crude prevalence of VI was 8.81% (95% CI:8.01 – 9.67). This included MiVI (2.87%; 95% CI:2.41 – 3.40), MVI (4.6%; 95% CI:4.06 – 5.29), SVI (0.60%; 95% CI:0.40 –

For peer review only - http://bmjopen.bmj.com/site/about/guidelines.xhtml

0.87), and blindness (0.69%; 95% CI: 0.47 – 0.97). Age and gender adjusted prevalence was 7.15% (95% CI: 6.80 – 8.32) (Table 2).

Table 2: Crude prevalence and age and gender adjusted prevalence of visual impairment (VI).

	Crude prevalence (95% Confidence Intervals)	Age and gender adjusted prevalence (95% Confidence Intervals)
Mild VI	2.87 (2.41 - 3.40)	2.37 (2.00 - 2.90)
Moderate VI	4.64 (4.06 - 5.29)	4.03 (3.50 - 4.64)
Severe VI	0.60 (0.40 - 0.87)	0.55 (0.35 - 0.80)
Blind	0.69 (0.47 - 0.97)	0.57 (0.37 - 0.82)
**All VI**	**8.81 (8.01 - 9.67)**	**7.51 (6.80 - 8.32)**

Risk factors for VI

On univariate analysis, the prevalence of VI was highest (41.2%) in the oldest age group. Though the prevalence of VI did not vary with gender (p=0.11), it was significantly higher among those with no formal education (16.4% versus 2.1%; p<0.01). The prevalence of VI was also higher among those who self-reported hypertension (18.2% versus 7.9%; p<0.01) and diabetes (27.1% versus 8.3%; p<0.01).

The multiple regression analysis showed that the odds for VI increased with increasing age. Compared to the participants aged 16-29 years, the odds for VI were 6.78 (95% CI: 3.46–13.29) for the 50-59 age group, 6.7 (95% CI: 3.4–13.2) for the 60-69 age group, and 33.7 (95% CI: 17.19–66.37) in the above 70 and older age group. The participants with no formal education had higher odds for VI compared to those with formal education (OR: 2.75; 95% CI: 1.90–3.97). Similarly, the participants with a history of diabetes had higher odds for VI (OR: 1.83; 95% CI: 1.16–2.89). Women had lower odds for VI compared to men (OR: 0.64; 95% CI: 0.51–0.82). Hypertension was not associated with VI (p=0.321) (Table 3).

For peer review only - http://bmjopen.bmj.com/site/about/guidelines.xhtml

Table 3: Effects of socio-demographic variables in visual impairment (multiple logistic regression analysis)

	Adjusted Odds Ratio (95% Confidence Intervals)	p-value
**Age group (years)**		
16- 29	Reference	
30-39	0.93 (0.4 - 2.11)	0.865
40-49	2.02 (0.99 - 4.13)	0.053
50-59	6.78 (3.46 -13.29)	<0.01
60-69	18.83 (9.6 -36.87)	<0.01
>=70	33.77 (17.19 - 66.37)	<0.01
**Gender**		
Male	Reference	
Female	0.64 (0.51- 0.82)	<0.01
**Education**		
Any education	Reference	
No education	2.75 (1.90 – 3.97)	<0.01
**Hypertension**		
No	Reference	
Yes	0.85 (0.62 - 1.17)	0.321
**Diabetes**		
No	Reference	
Yes	1.83 (1.16 - 2.89)	0.01

Causes of VI

Overall, uncorrected refractive errors (49.5%) were the leading cause of VI, followed by cataracts (40.2%) and posterior segment diseases (4.9%). Uncorrected refractive errors were the leading cause of moderate and severe VI, and cataract were the leading cause of blindness. Uncorrected refractive errors were the leading cause of VI in the younger age group (16-59 years), and cataract were the leading cause of VI in the older age group (60 years and older) (Figure 1).

Temporal trends in VI.

The data of individuals aged ≥40 years examined in the 2014 and 2021-2022 studies were analyzed to capture the trends in the prevalence of VI over time.[14] In total, 2,974 and 2,375 participants aged ≥40 years were examined in the 2014 and 2021-2022 studies, respectively. The mean age (±standard deviation) of the participants was higher in the 2022 study (55.0 ±11.0 years versus 51.7±9.9 years; p<0.05). Similarly, a lesser proportion of men were examined in the 2022 study (42.5% versus 45.6%; p=0.03). Overall, the prevalence of VI declined by 19.3% compared to the 2014 baseline study (20.2% to 16.3%; p<0.01). In

For peer review only - http://bmjopen.bmj.com/site/about/guidelines.xhtml

terms of categories of VI, MiVI declined by 21.9% (from 6.4% to 5.0%; p=0.03), MSVI declined by 15.1% (from 11.9% to 10.1%; p=0.04), and blindness declined by 36.8% (from 1.9% to 1.6%).

DISCUSSION

We have reported on the prevalence and causes of VI among the adult population in the Nirmal district of Telangana using the extended RAVI methodology. The conventional RAAB and RAVI methods include individuals aged ≥50 years and ≥40 years, respectively. In contrast, the extended RAVI methodology used in this study included anyone ≥16 years. While it is advantageous to only include the older population to minimize the sample size and use of resources, the data on VI is not readily available in the younger age groups. Data on all ages is essential to plan universal eye health initiatives in the region. The extended RAVI is an attempt to provide comprehensive information on the prevalence of VI in the complete adult population in this region. The data from this study can supplement the data from school eye health programs, providing a complete picture of the entire population, other than children under five years. In addition, we used the revised WHO definitions in this study for cross-comparison with other studies done in India and other regions of the world.

The Andhra Pradesh Eye Disease Study (APEDS) conducted between 1996 and 2000 was the only population-based cross-sectional study that included the population of all ages. The prevalence of Moderate VI, Severe VI, and blindness were 10.1%, 2.3%, and 2.3%, respectively. [19,20] Using similar definitions, the prevalence of Moderate VI, Severe VI, and blindness in this study were 4.6%, 0.60%, and 0.69%, respectively.[19,21] The prevalence of Mild VI is not reported in APEDS. Despite a difference in the age groups between the studies, a lower prevalence in this study indicates a decline in the prevalence of VI in this region over the last three decades. Such a secular trend of decline in VI has been reported from various locations, suggesting an improvement in the availability and uptake of eye care services in this region.

Both APEDS and the current study had a higher prevalence of VI among the older participants, which is consistent across all the studies conducted in this region.[19,21] In this study, though the prevalence did not vary with gender, women had lower odds for VI, which is contrary to the APEDS study, where women had a higher prevalence of VI. This difference could be attributed to availability, acceptability, and a higher uptake of eye care services

For peer review only - http://bmjopen.bmj.com/site/about/guidelines.xhtml

among women. A higher prevalence of VI was also noted among those with lower levels of education, which is similar to other studies in this region.[11,14,19,21] The higher visual needs and availability of resources for eye examinations and treatment might have attributed to a lower prevalence of VI among those with higher levels of education. The participants who self-reported diabetes had a higher prevalence of VI in this study, which might be caused by the earlier incidence of cataract secondary to diabetes and other refractive changes in the eye.

Several studies have reported the prevalence of VI using the RAVI methodology among participants aged ≥40 years in Andhra Pradesh, Telangana, and other parts of India.[14,15, 22,23,17-24] The prevalence of VI ranges from 8.7% in Tripura,[24] 10% in Krishna district in Andhra Pradesh,[15] 11.4% in Delhi,[22] 12.8% in Ganjam and Khordha districts in Odisha,[23] 12.8% in Akividu region West Godavari and Krishna districts,[17] and 13.7% in Mahbubnagar and Adilabad districts in Telangana.[14] These are comparable to the 11.3% found in the current study.

Uncorrected refractive errors and cataract remain the leading causes of VI.[11,14,17, 24-26] Similarly to APEDS, uncorrected refractive errors are the leading causes of VI in the older age groups compared to the younger age groups.[19,21] Moreover, similar to other studies, cataract was more common among those with severe grades of VI.[11,14,17, 25,26] As both these conditions are manageable with cost-effective interventions, strategies are needed to reach these communities and provide eye care.

The temporal trends on the prevalence of VI have been reported from Telangana.[26-28] In an earlier paper, we compared two studies conducted using the same methodology and geographical locations, Khammam and Warangal districts, that included identical age groups.[26] There was a 2.5% decline in VI in Khammam district, but it remained stable in Warangal district over five years.[26] In this study, we observed a 19% decline in VI compared to the study conducted in 2014, which is an annual decline of 2%. This decline could be attributed to increased availability and uptake of eye care services in this region. However, due to the absence of a control arm, the role of secular trends resulting in the decline of VI cannot be ruled out.

For peer review only - http://bmjopen.bmj.com/site/about/guidelines.xhtml

The Nirmal district (erstwhile Adilabad district) has witnessed a few epidemiological studies over the years. The Andhra Pradesh Eye Disease Study (APEDS) was conducted in 1997-98, followed by Rapid Assessment of Cataract Surgical Services (RACSS), a couple of RAVI studies in 2014, and the current study in 2022 (Table 4). Among those aged ≥40 years, the prevalence of VI (<6/18 definition) was 35.2% in APEDS, which dropped to 13.7% in the 2014 RAVI study. The prevalence declined further to 9.7% in the current study. Among those aged 50 years and older, the prevalence of VI (<6/18 definition) was 50.7% in APEDS, which dropped to 21.6% in RACSS conducted in 2006-07, and remained stable in the 2014 RAVI study, declining to 16.2% in the current study. As different protocols have been used over the years, direct comparisons are limited by the different measurement methods. Nevertheless, VI is declining in this region, as indicated by the two recent RAVI studies using identical protocols.

Table 4: Prevalence of visual impairment in Nirmal district by reported various studies.

	**Year**	**Sample size**	**Moderate Visual Impairment** (Presenting visual acuity worse than 6/18 to 6/60)	**Severe Visual Impairment** (Presenting visual acuity worse than 6/60 to 3/60)	**Blindness** (Presenting visual acuity worse than 3/60)	**Total Visual Impairment** (Presenting visual acuity worse than 6/18)
		**n**	**%**	**%**	**%**	**%**
**≥40 years**						
APEDS*	1997-98	840	28.5	1.8	5.0	35.2
RAVI †	2014	2974	10.4	1.5	1.9	13.7
RAVI †	2022	2392	8.8	0.4	0.6	9.7
**≥50 years**						
APEDS *	1997-98	521	40.3	2.9	7.5	50.7
RACSS ∫	2006-07	2160	13.6	4.8	3.2	21.6
RAVI †	2014	1550	16.6	2.3	3.0	21.9
RAVI †	2022	1491	13.4	1.1	1.7	16.2

** Andhra Pradesh Eye Disease Study. †Rapid Assessment of Visual Impairment. ∫ Rapid Assessment of Cataract Surgical Services.*

A good response rate, a randomly selected population-based sample, the use of an updated WHO definition of VI, and the inclusion of participants of wider age groups are the strengths of this study. After APEDS, this is the major study to report VI in the adult population in this region. However, a few studies have reported the VI in older age groups during this gap of

For peer review only - http://bmjopen.bmj.com/site/about/guidelines.xhtml

two decades. A higher proportion of women were examined in this study, which could be due to the migration of men to urban areas in search of work, a common occurrence in Telangana. The previous studies in this region also show a female preponderance.[11,14] Therefore, the overrepresentation of women could have overestimated the prevalence of VI in our study.

In conclusion, a significant burden of VI is observed in the region. However, the declining trend in the prevalence of VI suggests that the eye care services in the region are improving. This study can be a guide for more focused efforts to address vision loss and achieve universal eye health in this region. Also, there is a need for integration and eye care services with primary health care as people with diabetes had a higher prevalence of VI. VI impedes the attainment of sustainable development goals and the overall quality of life. Efforts to address vision loss might have a ripple effect on the overall health and well-being of individuals, families, and communities, contributing towards sustainable development goals.

Acknowledgements: The authors thank the vision technicians (Sunil Anant Doke, Vaibhav Kumar Dave, Jalindar Kumar Doke, Degaon Ganesh, Gangadharolla Vamshi) and community eye health team (Yedla Sandeep, Akaram Chinnaiah, Laxman Sunkari, Gunde Rao Rahul, Ramnapoina Mahesh) for the assistance provided in data collection. The authors also thank Sandeep Dayal, Thirupathi Reddy Kumbham, Shekhar Konegari and Saptak Banerjee for monitoring the study. Abhinav Sekar is acknowledged for the language inputs on earlier versions of our manuscript. The authors thank the volunteers for their participation in the study.

Contributors: SM conceived the idea, designed and conducted the study, analyzed the data and wrote the manuscript. AC was involved in data collection and quality control. VKY, RSV, RCK, RN reviewed the earlier version of the manuscripts and provided the intellectual inputs.

Funding Support: This work was supported by the DBT Wellcome Trust India Alliance Clinical Research Centre Grant [grant number IA/CRC/19/1/610010] awarded to Dr Raja Narayanan and Hyderabad Eye Research Foundation, India.

Competing Interests: The authors report no conflicts of interest and have no proprietary interest in any of the materials mentioned in this article.

Data sharing statement: No additional data are available.

Ethics Approval: The study protocol was approved by the Institutional Review Board (IRB) of Hyderabad Eye Research Foundation, L V Prasad Eye Institute (LVPEI) (Reference ID: LEC-08173). This study was conducted in accordance with the tenets of the Declaration of

For peer review only - http://bmjopen.bmj.com/site/about/guidelines.xhtml

Helsinki. Written informed consent was obtained from all the participants (or the legal guardian if the age of the participant is less than 18 years) before enrolment in the study.

REFERENCES

1. Bourne R, Steinmetz JD, Flaxman S, et al. Trends in prevalence of blindness and distance and near vision impairment over 30 years: an analysis for the Global Burden of Disease Study. The Lancet Global Health 2021;**9**(2):e130-e43 doi: https://doi.org/10.1016/S2214-109X(20)30425-3[published Online First: Epub Date]|.

2. Steinmetz JD, Bourne RRA, Briant PS, et al. Causes of blindness and vision impairment in 2020 and trends over 30 years, and prevalence of avoidable blindness in relation to VISION 2020: the Right to Sight: an analysis for the Global Burden of Disease Study. The Lancet Global Health 2021;**9**(2):e144-e60 doi: 10.1016/S2214-109X(20)30489-7[published Online First: Epub Date]|.

3. Marmamula S, Keeffe JE, Rao GN. Rapid assessment methods in eye care: An overview. Indian J Ophthalmol 2012;**60**(5):416-22 doi: IndianJOphthalmol_2012_60_5_416_100539 [pii]
10.4103/0301-4738.100539[published Online First: Epub Date]|.

4. McCormick I, Mactaggart I, Resnikoff S, et al. Eye health indicators for universal health coverage: results of a global expert prioritisation process. Br J Ophthalmol 2022;**106**(7):893-901 doi: 10.1136/bjophthalmol-2020-318481[published Online First: Epub Date]|.

5. Mactaggart I, Limburg H, Bastawrous A, Burton MJ, Kuper H. Rapid Assessment of Avoidable Blindness: looking back, looking forward. Br J Ophthalmol. 2019;**103**:1549-52

6. Marmamula S, Khanna RC, Narsaiah S, Shekhar K, Rao GN. Prevalence of spectacle use in Andhra Pradesh, India: Rapid Assessment of Visual Impairment (RAVI) project. Clin Exp Ophthalmol 2013 doi: 10.1111/ceo.12160[published Online First: Epub Date]|.

7. Marmamula S, Madala SR, Rao GN. Rapid assessment of visual impairment (RAVI) in marine fishing communities in South India--study protocol and main findings. BMC Ophthalmol 2011;**11**:26 doi: 10.1186/1471-2415-11-26[published Online First: Epub Date]|.

8. Marmamula S, Madala SR, Rao GN. Prevalence of uncorrected refractive errors, presbyopia and spectacle coverage in marine fishing communities in South India:

For peer review only - http://bmjopen.bmj.com/site/about/guidelines.xhtml

Rapid Assessment of Visual Impairment (RAVI) project. Ophthalmic Physiol Opt 2012;**32**(2):149-55 doi: 10.1111/j.1475-1313.2012.00893.x[published Online First: Epub Date]|.

9. Marmamula S, Narsaiah S, Shekhar K, Khanna RC. Visual Impairment among Weaving Communities in Prakasam District in South India. PLoS One 2013;**8**(2):e55924 doi: 10.1371/journal.pone.0055924[published Online First: Epub Date]|.
10. Marmamula S, Narsaiah S, Shekhar K, Khanna RC. Presbyopia, spectacles use and spectacle correction coverage for near vision among cloth weaving communities in Prakasam district in South India. Ophthalmic Physiol Opt 2013 doi: 10.1111/opo.12079[published Online First: Epub Date]|.
11. Marmamula S, Narsaiah S, Shekhar K, Khanna RC, Rao GN. Visual Impairment in the South Indian State of Andhra Pradesh: Andhra Pradesh - Rapid Assessment of Visual Impairment (AP-RAVI) Project. PLoS One 2013;**8**(7):e70120 doi: 10.1371/journal.pone.0070120[published Online First: Epub Date]|.
12. Marmamula S, Yelagondula VK. The feasibility of combining rapid assessment studies for measuring the progress toward Universal Eye Health. Indian J Ophthalmol 2023;**71**(5):2296-97 doi: 10.4103/IJO.IJO_2996_22[published Online First: Epub Date]|.
13. Marmamula S, Modepalli SB, Kumbham TR, Challa R, Keeffe JE. Prevalence of disabilities and non-communicable diseases in an elderly population in the Telangana state, India: a population-based cross-sectional study. BMJ open 2021;**11**(2):e041755 doi: 10.1136/bmjopen-2020-041755[published Online First: Epub Date]|.
14. Marmamula S, Khanna RC, Kunkunu E, Rao GN. Population-based assessment of prevalence and causes of visual impairment in the state of Telangana, India: a cross-sectional study using the Rapid Assessment of Visual Impairment (RAVI) methodology. BMJ open 2016;**6**(12):e012617 doi: 10.1136/bmjopen-2016-012617[published Online First: Epub Date]|.
15. Marmamula S, Khanna RC, Narsaiah S, Shekhar K, Rao GN. Prevalence of spectacles use in Andhra Pradesh, India: rapid assessment of visual impairment project. Clin Exp Ophthalmol 2014;**42**(3):227-34 doi: 10.1111/ceo.12160[published Online First: Epub Date]|.
16. Marmamula S, Khanna RC, Shekhar K, Rao GN. A population-based cross-sectional study of barriers to uptake of eye care services in South India: the Rapid Assessment

For peer review only - http://bmjopen.bmj.com/site/about/guidelines.xhtml

of Visual Impairment (RAVI) project. BMJ open 2014;**4**(6):e005125 doi: 10.1136/bmjopen-2014-005125[published Online First: Epub Date]|.

17. Marmamula S, Yelagondula VK, Khanna RC, et al. A Population-Based Cross-Sectional Study of Visual Impairment in West Godavari and Krishna Districts in Andhra Pradesh: Akividu Visual Impairment Study (AVIS). Ophthalmic Epidemiol 2022;**29**(4):411-16 doi: 10.1080/09286586.2021.1946829[published Online First: Epub Date]|.
18. Flaxman SR, Bourne RRA, Resnikoff S, et al. Global causes of blindness and distance vision impairment 1990-2020: a systematic review and meta-analysis. Lancet Glob Health 2017;**5**(12):e1221-e34 doi: 10.1016/S2214-109X(17)30393-5[published Online First: Epub Date]|.
19. Dandona L, Dandona R, Srinivas M, et al. Blindness in the Indian state of Andhra Pradesh. Invest Ophthalmol Vis Sci 2001;**42**(5):908-16
20. Dandona R, Dandona L, Srinivas M, et al. Moderate visual impairment in India: the Andhra Pradesh Eye Disease Study. Br J Ophthalmol 2002;**86**(4):373-7
21. Dandona L, Dandona R, Naduvilath TJ, et al. Burden of moderate visual impairment in an urban population in southern India. Ophthalmology 1999;**106**(3):497-504
22. Gupta N, Vashist P, Malhotra S, Senjam SS, Misra V, Bhardwaj A. Rapid assessment of visual impairment in urban population of Delhi, India. PLoS One 2015;**10**(4):e0124206 doi: 10.1371/journal.pone.0124206[published Online First: Epub Date]|.
23. Bhardwaj A, Vashist P, Singh Senjam S, Gupta V, Gupta N, Manna S. Rapid Assessment of Avoidable Visual Impairment in Two Coastal Districts of Eastern India for Determining Effective Coverage: A Cross-Sectional Study. J Ophthalmic Vis Res 2023;**18**(2):182-91 doi: 10.18502/jovr.v18i2.13185[published Online First: Epub Date]|.
24. Marmamula S, Yellapragada S, Khanna RC. Population Based Assessment of Prevalence and Causes of Vision Impairment in the North-eastern State of Tripura, India - The Tripura Eye Survey. Ophthalmic Epidemiol 2020;**27**(4):283-88 doi: 10.1080/09286586.2020.1731833[published Online First: Epub Date]|.
25. Senjam SS, Vashist P, Gupta N, et al. Prevalence of visual impairment due to uncorrected refractive error: Results from Delhi-Rapid Assessment of Visual Impairment Study. Indian J Ophthalmol 2016;**64**(5):387-90 doi: 10.4103/0301-4738.185614[published Online First: Epub Date]|.

For peer review only - http://bmjopen.bmj.com/site/about/guidelines.xhtml

26. Marmamula S, Khanna RC, Yellapragada S, Challa R, Mohd J, Rao GN. Temporal trends in the prevalence and causes of visual impairment in the South Indian state of Telangana: a population-based cross-sectional study. BMJ open 2019;**9**(7):e029114 doi: 10.1136/bmjopen-2019-029114[published Online First: Epub Date]|.

27. Khanna RC, Marmamula S, Krishnaiah S, Giridhar P, Chakrabarti S, Rao GN. Changing trends in the prevalence of blindness and visual impairment in a rural district of India: Systematic observations over a decade. Indian J Ophthalmol 2012;**60**(5):492-7 doi: IndianJOphthalmol_2012_60_5_492_100560 [pii]
10.4103/0301-4738.100560[published Online First: Epub Date]|.

28. Marmamula S, Keeffe JE, Narsaiah S, Khanna RC, Rao GN. Changing trends in the prevalence of visual impairment, uncorrected refractive errors and use of spectacles in Mahbubnagar district in South India. Indian J Ophthalmol 2013;**61**(12):755-8 doi: 10.4103/0301-4738.121149[published Online First: Epub Date]|.

Figure 1: Causes of visual impairment across the age groups.

For peer review only - http://bmjopen.bmj.com/site/about/guidelines.xhtml

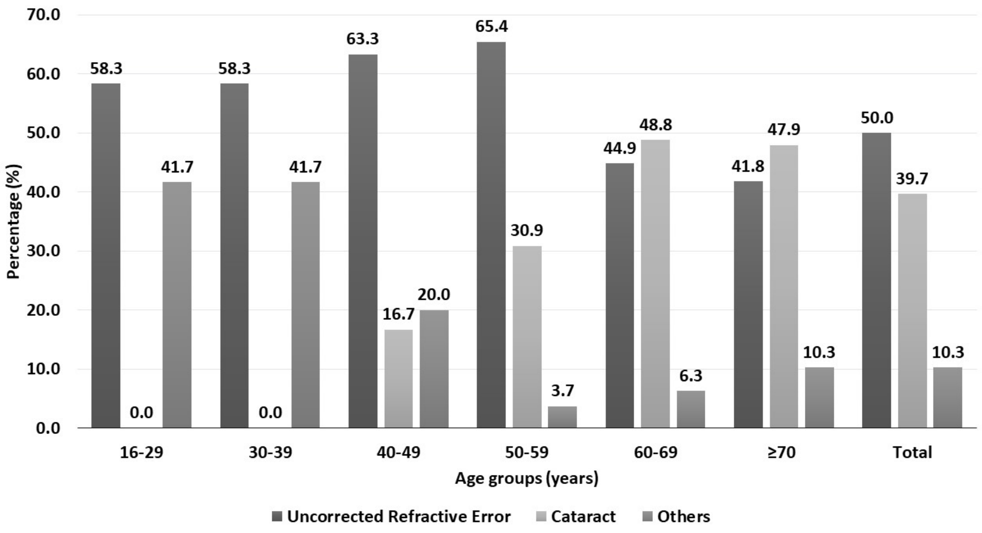

Causes of visual impairment across the age groups

338x190mm (96 x 96 DPI)

ID				

RAPID ASSESSMENT OF VISUAL IMPAIRMENT PROJECT – DATA COLLECTION FORM

Section A – Demographic Informatio

Address:

Name ____ **Age** ____ **Mobile** ____

Status
Circle the codes

1	Examined
2	Not available after 2 visits
3	Refused

Gender

1	Male
2	Female

Education Level

0	No education
1	Primary school (class 1-5)
2	High school (class 6-10)
3	Intermediate (class 11-12)
4	College (undergraduate)
5	Advanced studies (PG etc)
6	Others, specify

Occupation

0	Unemployed
1	Teacher / clerical jobs
2	Driver
3	Shop Keeper / Business
4	Labourer – Agriculture
5	Labourer – Other
6	Beedi rolling
7	Stopped working due to poor vision
8	Home duties only
9	Retired / stopped worked due to old age
10	Others, specify

Section B – Spectacle Information

Present Glasses

0	No glasses
1	Yes

Amount paid ____

Glasses - Type

0	No glasses
1	SV - Distance
2	SV- Near
3	Bifocals

Provider

0	No Glasses
1	LVP Vision Centre
2	LVP Service centre
3	Private Eye doctor / Clinic
4	Directly from the local optical shop
5	Govt. in a camp for no cost

Section C – Surgery Information and Systemic Conditions (Please enter the codes)

	RE	LE
**When was surgery done**		
**Costs of surgery**		

0	Not applicable
1	Free / Nonpaying
2	Paying **Amount**

	RE	LE
**Place of surgery**		

0	Not applicable
1	Eye camp
2	NGO hospital
3	Private hospital
4	Government hospital

Systemic Condition ***(Enter the code as appropriate)***

Condition	Duration (yrs)

Codes: 0= None, 1=HTN, 2= DM, 3= Heart disease, 4= Asthma, 5=Other, specify

For peer review only

Section D – Visual Acuity and Clinical Examination ***(Enter the code as appropriate)***

**Visual acuity**	**RE**	**LE**	**BE**
Unaided - Distance			
Pinhole - Distance			
(Only if <6/12)			
Aided – Distance*			
Pinhole - Distance			
**PRESENTING VA (PVA)**			

**Lens status**		**RE**	**LE**
1	Normal lens		
2	Obvious lens opacity/cataract		
3	Aphakia		
4	Pseudophakia		
5	No view of lens, why		

Unaided – Near (Binocular)	
Aided - Near	
Add power used	+
Near vision with addition	

* *Record aided vision with +10 for aphakia*

(Codes: cf 3 mts=8; cf 2mts=9; cf 1m=10 PLPR=11; NOPL=12)

**Other Major Finding**			
0	None		
1	Corneal scar		
2	Pterygium		
3	Posterior capsular opacification		
4	Others		

Section E – Primary causes of Visual Impairment ***(Enter the code as appropriate)***

	**Principal cause of presenting vision <6/12**
0	No visual impairment
1	Refractive Error
2	Uncorrected aphakia
3	Cataract
4	Surgery related complications
5	Corneal opacity
6	Phthisis or absent globe
7	Glaucoma
8	Posterior segment disorders
9	Others, specify

**RE**	**LE**	**BIN**

(BIN=least of the two)

Please Note:
Presenting VA= Aided VA if subjects has glasses;
Presenting VA= Unaided VA if subject has no glasses

Name of the examiner: ______________________

Signature/Date: ____________________________

For peer review only

STROBE 2007 (v4) Statement—Checklist of items that should be included in reports of *cross-sectional studies*

Section/Topic	Item #	Recommendation	Reported on page #
**Title and abstract**	1	(*a*) Indicate the study's design with a commonly used term in the title or the abstract	1
		(*b*) Provide in the abstract an informative and balanced summary of what was done and what was found	1
**Introduction**			
Background/rationale	2	Explain the scientific background and rationale for the investigation being reported	3
Objectives	3	State specific objectives, including any pre-specified hypotheses	3
**Methods**			
Study design	4	Present key elements of study design early in the paper	3
Setting	5	Describe the setting, locations, and relevant dates, including periods of recruitment, exposure, follow-up, and data collection	4
Participants	6	(*a*) Give the eligibility criteria, and the sources and methods of selection of participants	4
Variables	7	Clearly define all outcomes, exposures, predictors, potential confounders, and effect modifiers. Give diagnostic criteria, if applicable	4,5
Data sources/ measurement	8*	For each variable of interest, give sources of data and details of methods of assessment (measurement). Describe comparability of assessment methods if there is more than one group	4,5
Bias	9	Describe any efforts to address potential sources of bias	NA
Study size	10	Explain how the study size was arrived at	na
Quantitative variables	11	Explain how quantitative variables were handled in the analyses. If applicable, describe which groupings were chosen and why	6
Statistical methods	12	(*a*) Describe all statistical methods, including those used to control for confounding	6
		(*b*) Describe any methods used to examine subgroups and interactions	6
		(*c*) Explain how missing data were addressed	NA
		(*d*) If applicable, describe analytical methods taking account of sampling strategy	NA
		(*e*) Describe any sensitivity analyses	NA
**Results**			

For peer review only

Participants	13*	(a) Report numbers of individuals at each stage of study—eg numbers potentially eligible, examined for eligibility, confirmed eligible, included in the study, completing follow-up, and analysed	NA
		(b) Give reasons for non-participation at each stage	NA
		(c) Consider use of a flow diagram	NA
Descriptive data	14*	(a) Give characteristics of study participants (eg demographic, clinical, social) and information on exposures and potential confounders	7
		(b) Indicate number of participants with missing data for each variable of interest	NA
Outcome data	15*	Report numbers of outcome events or summary measures	7
Main results	16	(*a*) Give unadjusted estimates and, if applicable, confounder-adjusted estimates and their precision (eg, 95% confidence interval). Make clear which confounders were adjusted for and why they were included	7,8,9
		(*b*) Report category boundaries when continuous variables were categorized	6
		(*c*) If relevant, consider translating estimates of relative risk into absolute risk for a meaningful time period	6
Other analyses	17	Report other analyses done—eg analyses of subgroups and interactions, and sensitivity analyses	7,8,9
**Discussion**			
Key results	18	Summarise key results with reference to study objectives	10
Limitations	19	Discuss limitations of the study, taking into account sources of potential bias or imprecision. Discuss both direction and magnitude of any potential bias	12,13
Interpretation	20	Give a cautious overall interpretation of results considering objectives, limitations, multiplicity of analyses, results from similar studies, and other relevant evidence	12,13
Generalisability	21	Discuss the generalisability (external validity) of the study results	13
**Other information**			
Funding	22	Give the source of funding and the role of the funders for the present study and, if applicable, for the original study on which the present article is based	13

*Give information separately for cases and controls in case-control studies and, if applicable, for exposed and unexposed groups in cohort and cross-sectional studies.

Note: An Explanation and Elaboration article discusses each checklist item and gives methodological background and published examples of transparent reporting. The STROBE checklist is best used in conjunction with this article (freely available on the Web sites of PLoS Medicine at http://www.plosmedicine.org/, Annals of Internal Medicine at http://www.annals.org/, and Epidemiology at http://www.epidem.com/). Information on the STROBE Initiative is available at www.strobe-statement.org.

For peer review only

BMJ Open

A cross-sectional study of prevalence, causes, and trends in visual impairment in Nirmal District, Telangana, India - Nirmal Eye Evaluation for Trends study.

Journal:	*BMJ Open*
Manuscript ID	bmjopen-2023-083199.R2
Article Type:	Original research
Date Submitted by the Author:	24-Apr-2024
Complete List of Authors:	Marmamula, Srinivas; LV Prasad Eye Institute, Gullapalli Pratibha Rao International Centre for Advancement of Rural Eye care; University of New South Wales<br>Chinya, Aritra; LV Prasad Eye Institute<br>Yelagondula, Vijay; LV Prasad Eye Institute, Allen Foster Community Eye Health Research Centre, ICARE<br>Varda, Rajashekar; LV Prasad Eye Institute<br>Khanna, Rohit; LV Prasad Eye Institute<br>Narayanan, Raja; LV Prasad Eye Institute, Vitreo-retina & uveitis
**Primary Subject Heading**:	Ophthalmology
Secondary Subject Heading:	Epidemiology, Public health
Keywords:	EPIDEMIOLOGIC STUDIES, PUBLIC HEALTH, Observational Study

SCHOLARONE™
Manuscripts

I, the Submitting Author has the right to grant and does grant on behalf of all authors of the Work (as defined in the below author licence), an exclusive licence and/or a non-exclusive licence for contributions from authors who are: i) UK Crown employees; ii) where BMJ has agreed a CC-BY licence shall apply, and/or iii) in accordance with the terms applicable for US Federal Government officers or employees acting as part of their official duties; on a worldwide, perpetual, irrevocable, royalty-free basis to BMJ Publishing Group Ltd ("BMJ") its licensees and where the relevant Journal is co-owned by BMJ to the co-owners of the Journal, to publish the Work in this journal and any other BMJ products and to exploit all rights, as set out in our licence.

The Submitting Author accepts and understands that any supply made under these terms is made by BMJ to the Submitting Author unless you are acting as an employee on behalf of your employer or a postgraduate student of an affiliated institution which is paying any applicable article publishing charge ("APC") for Open Access articles. Where the Submitting Author wishes to make the Work available on an Open Access basis (and intends to pay the relevant APC), the terms of reuse of such Open Access shall be governed by a Creative Commons licence – details of these licences and which Creative Commons licence will apply to this Work are set out in our licence referred to above.

Other than as permitted in any relevant BMJ Author's Self Archiving Policies, I confirm this Work has not been accepted for publication elsewhere, is not being considered for publication elsewhere and does not duplicate material already published. I confirm all authors consent to publication of this Work and authorise the granting of this licence.

For peer review only

ORIGINAL RESEARCH

A cross-sectional study of prevalence, causes, and trends in visual impairment in Nirmal District, Telangana, India - Nirmal Eye Evaluation for Trends study.

Srinivas Marmamula, Ph.D.[1,2,3,4] Aritra Chinya, MPH,[1,2] Vijay Kumar Yelagondula, M.Optom,[2] Rajashekar Varada, MPhil,[1]

Rohit C Khanna, MD[1,4] Raja Narayanan, MS[3,5,6]

[1] Allen Foster Community Eye Health Research Centre, Gullapalli Pratibha Rao International Centre for Advancement of Rural Eye care, L V Prasad Eye Institute, Hyderabad, India

[2] Brien Holden Institute of Optometry and Vision Science, L V Prasad Eye Institute, Hyderabad, India

[3] Wellcome Trust / Department of Biotechnology India Alliance, L V Prasad Eye Institute, Hyderabad, India

[4] School of Optometry and Vision Science, University of New South Wales, Sydney, Australia

[5] Anant Bajaj Retina Institute, L V Prasad Eye Institute, Hyderabad, India

[6] Suven Clinical Research Centre, L V Prasad Eye Institute, Hyderabad, India

Running Title: Visual Impairment in Telangana, India

Keywords: Visual impairment, rapid assessment studies, trends, Universal Eye Health, India

Corresponding author:

Dr. Srinivas Marmamula, Gullapalli Pratibha Rao International Centre for Advancement of Rural Eye care, L V Prasad Eye Institute, Hyderabad, India. 500034

Email: sri.marmamula@lvpei.org

Total number of words:3252 words excluding title page, abstract, acknowledgements, references, tables and figure legends

For peer review only - http://bmjopen.bmj.com/site/about/guidelines.xhtml

ABSTRACT

Objective: To determine the prevalence, causes, and risk factors associated with visual impairment (VI) in the Nirmal district of Telangana, India, using extended Rapid Assessment of Visual Impairment (RAVI) methodology.

Design: Cross-sectional study

Setting: Community setting

Participants: Participants aged ≥16 years were enumerated from 90 randomly selected clusters and 4629/5400 (85.7%) participants were examined. Presenting visual acuity (VA) was assessed using a Snellen chart with E optotypes at a six-meter distance was recorded. Near vision was assessed binocularly using an N notation chart with tumbling E optotypes at a 40 cm distance. An anterior segment examination and distance direct ophthalmoscopy at 50 cm were also performed, and non-mydriatic fundus images were obtained. VI was defined as presenting VA worse than 6/12 in the better eye. The prevalence of VI in the current study was compared with a RAVI study conducted in 2014 to assess the trends in VI among those ≥40 years.

Primary outcome: Prevalence, causes and risk factors for VI.

Results: Among those examined, 55% were women, 53% had at least school-level education, 2.3% self-reported diabetes, and 8.7% self-reported hypertension. The prevalence of VI was 8.81% (95% CI:8.01-9.67). Overall, uncorrected refractive errors (49.5%) were the leading cause of VI, followed by cataracts (40.2%) and posterior segment diseases (4.9%). Among those aged ≥40 years, the prevalence of VI declined by 19.3% compared to the 2014 baseline study (20.2% to 16.3%; p<0.01).

Conclusion: The extended RAVI study conducted in the Nirmal district showed a considerable decline in the prevalence of VI. Targeted interventions are needed to provide adequate eye care for the high-risk groups in this district.

For peer review only - http://bmjopen.bmj.com/site/about/guidelines.xhtml

ARTICLE SUMMARY:

Strengths and limitations of the study

- Rapid assessments typically focus on participants aged ≥40 years. This study extends the rapid assessment methodology to include younger age groups (≥40 years) and provides estimates on the prevalence and causes of visual impairment.
- In addition to prevalence estimates, temporal trends in the prevalence of visual impairment are presented**.**
- As a randomly selected population-based sample was used, the results from the study can be extrapolated to the population in the region.
- The overrepresentation of women could have overestimated the prevalence of VI in our study.

For peer review only

For peer review only - http://bmjopen.bmj.com/site/about/guidelines.xhtml

INTRODUCTION

Over a billion people suffer from needless vision impairment (VI) globally, with cataracts and uncorrected refractive errors (URE) being the leading causes.[1,2] However, these conditions can be addressed using cost-effective interventions, such as spectacles and cataract surgery. Population-based data on the prevalence and causes of VI are essential to plan eye care service models to address this global problem. Though conventional epidemiological studies provide the data, they are often resource-intensive and need expertise to implement them. The rapid assessment methods are low-cost epidemiological tools that provide data on the prevalence and causes of VI using limited resources while being relatively easy to implement. In addition, these rapid assessments can be repeated at stipulated intervals to study the temporal trends in a given region.[3] Rapid assessment studies are even more important now, with WHO setting global targets for effective cataract surgical coverage and effective refractive error coverage as indicators to measure the progress toward Universal Eye Health.[4]

Rapid assessment studies initially focused on cataract alone; however, they were modified and evolved to cover other causes of VI, with an increasing focus on emerging eye conditions, such as diabetic retinopathy and refractive errors.[3,5] Rapid Assessment of Visual Impairment (RAVI) is an offshoot of multiple rapid assessment methods developed for eye care and has been used extensively in India and other countries.[6-11] STUDIES using the RAVI methodology focus on individuals aged 40 years and older. Recently, it has been modified to include younger individuals (≥16 years) and has been renamed as the extended RAVI methodology.[12] In addition, new tools have been added to collect data on systemic conditions and disabilities, helping to more holistic planning of holistic eye health programs.[5,13] The Nirmal Eye Evaluation for Trends is the first study to use extended RAVI. In this study, we report the prevalence, causes, and risk factors of VI in the Nirmal district and adjoining areas of Telangana, India. In addition to VI, this paper also compares the temporal trend in the prevalence of VI in this region using data from a previous study conducted in 2014.[14]

MATERIALS AND METHODS

Ethics Approval

The study protocol was approved by the Institutional Review Board (IRB) of Hyderabad Eye Research Foundation, L V Prasad Eye Institute (LVPEI) (Reference ID: LEC-08173). This

For peer review only - http://bmjopen.bmj.com/site/about/guidelines.xhtml

study was conducted in accordance with the tenets of the Declaration of Helsinki. Written informed consent was obtained from all the participants. For those aged less than 18 years of age, assent for the eye examination was obtained from the participant and written informed consent was obtained from the legal guardian.

Patient and public involvement

Patients and other members of the public were not involved in the design of the study.

Sampling strategy

Assuming a VI prevalence of 3.5% (presenting visual acuity worse than 6/12), allowing for a 95% confidence interval, a precision of 20%, a design effect of 1.6 for a predetermined cluster size of 60 participants, and a 20% non-response rate, the minimum sample size required was 5270, which was rounded up to 5400 (90 clusters) participants. A multi-stage cluster random sampling procedure with a compact segment sampling method was used, which has been described in previous reports.[14] The study area had a population of 0.5 million people and comprised ten sub-districts (mandal) from the Nirmal (eight) and Nizamabad (two) districts. The eye care needs of the study area were serviced by a secondary centre of LVPEI. Data were collected between November 2021 and March 2022.

Data collection

Three teams, comprising a vision technician and two community eye health workers, collected the data. They were supervised by a study coordinator (optometrist), who was also responsible for travel logistics and quality control. The examiners were trained to conduct the study procedures and document the findings. A reliability assessment was conducted before the study to assess the inter-observer agreement on visual acuity with a gold-standard senior optometrist. All examiners had a good agreement with the gold-standard optometrist (kappa 0.8 or more).

One of the three study teams visited participants at their homes and conducted eye examinations. The time of the visits was planned to maximize the availability of the participants at their households for examination. In each selected household, all the individuals who fulfilled the age criteria were documented, and all those who were available during the visit were examined. At least two attempts were made to examine those who were unavailable during the first visit, after which they were marked as unavailable.

For peer review only - http://bmjopen.bmj.com/site/about/guidelines.xhtml

Eye Examination protocol

A brief interview was conducted to collect personal and social-demographic information, such as age, education level, and systemic health conditions. (supplemental file - 1) Data related to the ocular history, including current and previous use of spectacles, use of eye drops, and details of any previous surgery, were also collected. Information regarding the barriers to the uptake of eye care services was also collected using a structured questionnaire.

The standard RAVI clinical examination was conducted after the interview, as described in previous studies.[11,15-17] In brief, the distance visual acuity (VA) was assessed using a standard Snellen chart with tumbling E optotypes at a distance of six meters. If a participant was unable to identify the letters in the first line of the chart, the distance between the participant and the chart was progressively reduced to three meters and then one meter till VA could be recorded. Unaided VA was recorded for all participants. Aided VA was recorded for participants using spectacles for correction. Aided VA was considered as the presenting VA for those with spectacles, and unaided VA was considered as the presenting VA. If the presenting VA was worse than 6/12, the VA was recorded using a multiple pinhole occluder. Near vision was assessed binocularly using the N notation chart at a fixed distance of 40 cm in ambient lighting conditions. The fixed distance was maintained using a string attached to the near vision chart. Both unaided and aided near vision were assessed if the participant reported spectacles use. Near vision was re-assessed using near addition lenses in a trial frame appropriate for that age among participants with near vision worse than N8.

An external eye examination was performed using a torchlight/portable slit lamp. The lens was assessed using distant direct ophthalmoscopy at about 50 cm distance in a shaded area (indoors), which was graded as normal, obvious lens opacity, aphakia, pseudophakia without posterior capsular opacification (PCO), or pseudophakia with PCO. If the lens could not be examined because of corneal opacities, phthisis bulbi, or absent globe, then it was documented in the data form. A non-mydriatic portable fundus camera (Visuscout 100 Handheld Fundus Camera, Carl Zeiss Meditec, USA) was used to capture retinal images. Two images, one optic disc-centred and another macula-centred, were captured for each eye. All the images were evaluated by experienced graders at L V Prasad Eye Institute. The participants with VI and those requiring other eye care services were referred to the nearest eye care facility for management.

For peer review only - http://bmjopen.bmj.com/site/about/guidelines.xhtml

The WHO categorizes visual impairment (VI) into four categories based on the presenting visual acuity in the better eye.[18] The four categories are as follows: Mild VI (MiVI - VA worse than 6/12 to 6/18), Moderate VI (MVI - VA worse than 6/18 to 6/60), Severe VI (SVI - worse than 6/60 to 3/60), and Blindness (VA worse than 3/60 to no perception of light). The case definitions for the causes of VI used in this study have been described in our previous publications.[11] In brief, uncorrected refractive error was defined as presenting VA <6/12, improving to 6/12 or better with pinhole. Cataract was defined as an opacity of the crystalline lens as seen with torchlight and obscuring the red reflex, partially or completely, on the distance direct ophthalmoscopy, resulting in a VA <6/12 that does not improve with pinhole. Posterior segment disease was considered as the cause of VI in cases where there was no media opacity and visual acuity did not improve with a pinhole. Posterior capsular opacification, and corneal opacities/edema after cataract surgery were marked as surgical complications. After the eye examination, the principal cause of VI was recorded for each eye separately, and then for the person. If there was more than one cause, the cause that was more easily treatable or correctable was marked as the main cause of visual impairment.

As this study was conducted during the pandemic, all COVID-19-related protocols were followed, including the use of masks (N-95) and visors at all times, frequent hand sanitization, and social distancing. All the team members were vaccinated before the start of the study. The equipment used, such as trial frames and multiple pinhole occluders, were disinfected with alcohol wipes/swabs after each use. The participants were offered hand sanitiser to clean their hands before starting the study procedures. The current health status of all the participants was enquired before the eye examinations.

Data Management

In the field, data were collected using paper forms. The forms were then transported to the data centre for entry into a Microsoft Access database. Data analyses were performed using the Stata Statistical Software for Windows, version 14 (StataCorp, College Station, TX). The prevalence estimates were adjusted to the age and gender distribution of the population for the year 2011, which have been presented with the 95% confidence intervals (CI). The demographic associations of VI with age, gender, education, and systemic conditions were assessed using multiple logistic regression models and adjusted odds ratios (OR) with 95% CI. A study using the conventional RAVI methodology was conducted in the same region in

For peer review only
For peer review only - http://bmjopen.bmj.com/site/about/guidelines.xhtml

2014.[14] The prevalence estimates from the current study were compared with the 2014 study to assess the trends in VI over time in this region.

RESULTS

Characteristics of the participants.

Of the 5400 participants included in this study from the 90 clusters, 4629 (85.7%) were examined. The mean age (±standard deviation) of the examined participants was similar to those not examined (42.5 (±16.6) years versus 42.0 (±16.5) years; p=0.38). A higher proportion of women were examined (55% versus 44%; p<0.01). Among those examined, 55% (n=2545) were women, 53% (2456) had at least school education, 2.3% (n=129) self-reported diabetes, and 8.7% (n=402) self-reported hypertension (Table 1).

Table 1: Visual impairment and demographic characteristics of the participants

	Total examined (n)	Number of participants with VI n (%)	p-value
**Age group (years)**			<0.01
16-29	1,244	12 (1.0)	
30-39	1,010	12 (1.2)	
40-49	899	30 (3.3)	
50-59	684	81 (11.8)	
60-69	440	127 (28.9)	
70 & above	352	146 (41.5)	
**Gender**			0.11
Male	2084	199 (9.5)	
Female	2545	209 (8.2)	
**Education level**			<0.01
No education	2173	356 (16.4)	
Any education	2456	52 (2.1)	
**Diabetes**			<0.01
Yes	129	35 (27.1)	
No	4500	373 (8.3)	
**Hypertension**			<0.01
Yes	402	73 (18.2)	
No	4227	335 (7.9)	
**Total**	**4629**	**408 (8.8)**	

The overall crude prevalence of VI was 8.81% (95% CI:8.01 – 9.67). This included MiVI (2.87%; 95% CI:2.41 – 3.40), MVI (4.6%; 95% CI:4.06 – 5.29), SVI (0.60%; 95% CI:0.40 –

For peer review only - http://bmjopen.bmj.com/site/about/guidelines.xhtml

0.87), and blindness (0.69%; 95% CI: 0.47 – 0.97). Age and gender adjusted prevalence was 7.15% (95% CI: 6.80 – 8.32) (Table 2).

Table 2: Crude prevalence and age and gender adjusted prevalence of visual impairment (VI).

	Crude prevalence (95% Confidence Intervals)	Age and gender adjusted prevalence (95% Confidence Intervals)
Mild VI	2.87 (2.41 - 3.40)	2.37 (2.00 - 2.90)
Moderate VI	4.64 (4.06 - 5.29)	4.03 (3.50 - 4.64)
Severe VI	0.60 (0.40 - 0.87)	0.55 (0.35 - 0.80)
Blind	0.69 (0.47 - 0.97)	0.57 (0.37 - 0.82)
**All VI**	**8.81 (8.01 - 9.67)**	**7.51 (6.80 - 8.32)**

Risk factors for VI

On univariate analysis, the prevalence of VI was highest (41.2%) in the oldest age group. Though the prevalence of VI did not vary with gender (p=0.11), it was significantly higher among those with no formal education (16.4% versus 2.1%; p<0.01). The prevalence of VI was also higher among those who self-reported hypertension (18.2% versus 7.9%; p<0.01) and diabetes (27.1% versus 8.3%; p<0.01).

The multiple regression analysis showed that the odds for VI increased with increasing age. Compared to the participants aged 16-29 years, the odds for VI were 6.78 (95% CI: 3.46–13.29) for the 50-59 age group, 6.7 (95% CI: 3.4–13.2) for the 60-69 age group, and 33.7 (95% CI: 17.19–66.37) in the above 70 and older age group. The participants with no formal education had higher odds for VI compared to those with formal education (OR: 2.75; 95% CI: 1.90–3.97). Similarly, the participants with a history of diabetes had higher odds for VI (OR: 1.83; 95% CI: 1.16–2.89). Women had lower odds for VI compared to men (OR: 0.64; 95% CI: 0.51–0.82). Hypertension was not associated with VI (p=0.321) (Table 3).

For peer review only - http://bmjopen.bmj.com/site/about/guidelines.xhtml

Table 3: Effects of socio-demographic variables in visual impairment (multiple logistic regression analysis)

	Adjusted Odds Ratio (95% Confidence Intervals)	p-value
**Age group (years)**		
16- 29	Reference	
30-39	0.93 (0.4 - 2.11)	0.865
40-49	2.02 (0.99 - 4.13)	0.053
50-59	6.78 (3.46 -13.29)	<0.01
60-69	18.83 (9.6 -36.87)	<0.01
>=70	33.77 (17.19 - 66.37)	<0.01
**Gender**		
Male	Reference	
Female	0.64 (0.51- 0.82)	<0.01
**Education**		
Any education	Reference	
No education	2.75 (1.90 – 3.97)	<0.01
**Hypertension**		
No	Reference	
Yes	0.85 (0.62 - 1.17)	0.321
**Diabetes**		
No	Reference	
Yes	1.83 (1.16 - 2.89)	0.01

Causes of VI

Overall, uncorrected refractive errors (49.5%) were the leading cause of VI, followed by cataracts (40.2%) and posterior segment diseases (4.9%). Uncorrected refractive errors were the leading cause of moderate and severe VI, and cataract were the leading cause of blindness. Uncorrected refractive errors were the leading cause of VI in the younger age group (16-59 years), and cataract were the leading cause of VI in the older age group (60 years and older) (Figure 1).

Temporal trends in VI.

The data of individuals aged ≥40 years examined in the 2014 and 2021-2022 studies were analyzed to capture the trends in the prevalence of VI over time.[14] In total, 2,974 and 2,375 participants aged ≥40 years were examined in the 2014 and 2021-2022 studies, respectively. The mean age (±standard deviation) of the participants was higher in the 2022 study (55.0 ±11.0 years versus 51.7±9.9 years; $p<0.05$). Similarly, a lesser proportion of men were examined in the 2022 study (42.5% versus 45.6%; $p=0.03$). Overall, the prevalence of VI declined by 19.3% compared to the 2014 baseline study (20.2% to 16.3%; $p<0.01$). In

For peer review only - http://bmjopen.bmj.com/site/about/guidelines.xhtml

terms of categories of VI, MiVI declined by 21.9% (from 6.4% to 5.0%; p=0.03), MSVI declined by 15.1% (from 11.9% to 10.1%; p=0.04), and blindness declined by 36.8% (from 1.9% to 1.6%).

DISCUSSION

We have reported on the prevalence and causes of VI among the adult population in the Nirmal district of Telangana using the extended RAVI methodology. The conventional RAAB and RAVI methods include individuals aged ≥50 years and ≥40 years, respectively. In contrast, the extended RAVI methodology used in this study included anyone ≥16 years. While it is advantageous to only include the older population to minimize the sample size and use of resources, the data on VI is not readily available in the younger age groups. Data on all ages is essential to plan universal eye health initiatives in the region. The extended RAVI is an attempt to provide comprehensive information on the prevalence of VI in the complete adult population in this region. The data from this study can supplement the data from school eye health programs, providing a complete picture of the entire population, other than children under five years. In addition, we used the revised WHO definitions in this study for cross-comparison with other studies done in India and other regions of the world.

The Andhra Pradesh Eye Disease Study (APEDS) conducted between 1996 and 2000 was the only population-based cross-sectional study that included the population of all ages. The prevalence of Moderate VI, Severe VI, and blindness were 10.1%, 2.3%, and 2.3%, respectively. [19,20] Using similar definitions, the prevalence of Moderate VI, Severe VI, and blindness in this study were 4.6%, 0.60%, and 0.69%, respectively.[19,21] The prevalence of Mild VI is not reported in APEDS. Despite a difference in the age groups between the studies, a lower prevalence in this study indicates a decline in the prevalence of VI in this region over the last three decades. Such a secular trend of decline in VI has been reported from various locations, suggesting an improvement in the availability and uptake of eye care services in this region.

Both APEDS and the current study had a higher prevalence of VI among the older participants, which is consistent across all the studies conducted in this region.[19,21] In this study, though the prevalence did not vary with gender, women had lower odds for VI, which is contrary to the APEDS study, where women had a higher prevalence of VI. This difference could be attributed to availability, acceptability, and a higher uptake of eye care services

For peer review only - http://bmjopen.bmj.com/site/about/guidelines.xhtml

among women. A higher prevalence of VI was also noted among those with lower levels of education, which is similar to other studies in this region.[11,14,19,21] The higher visual needs and availability of resources for eye examinations and treatment might have attributed to a lower prevalence of VI among those with higher levels of education. The participants who self-reported diabetes had a higher prevalence of VI in this study, which might be caused by the earlier incidence of cataract secondary to diabetes and other refractive changes in the eye.

Several studies have reported the prevalence of VI using the RAVI methodology among participants aged ≥40 years in Andhra Pradesh, Telangana, and other parts of India.[14,15, 22,23,17-24] The prevalence of VI ranges from 8.7% in Tripura,[24] 10% in Krishna district in Andhra Pradesh,[15] 11.4% in Delhi,[22] 12.8% in Ganjam and Khordha districts in Odisha,[23] 12.8% in Akividu region West Godavari and Krishna districts,[17] and 13.7% in Mahbubnagar and Adilabad districts in Telangana.[14] These are comparable to the 11.3% found in the current study.

Uncorrected refractive errors and cataract remain the leading causes of VI.[11,14,17, 24-26] Similarly to APEDS, uncorrected refractive errors are the leading causes of VI in the older age groups compared to the younger age groups.[19,21] Moreover, similar to other studies, cataract was more common among those with severe grades of VI.[11,14,17, 25,26] As both these conditions are manageable with cost-effective interventions, strategies are needed to reach these communities and provide eye care.

The temporal trends on the prevalence of VI have been reported from Telangana.[26-28] In an earlier paper, we compared two studies conducted using the same methodology and geographical locations, Khammam and Warangal districts, that included identical age groups.[26] There was a 2.5% decline in VI in Khammam district, but it remained stable in Warangal district over five years.[26] In this study, we observed a 19% decline in VI compared to the study conducted in 2014, which is an annual decline of 2%. This decline could be attributed to increased availability and uptake of eye care services in this region. However, due to the absence of a control arm, the role of secular trends resulting in the decline of VI cannot be ruled out.

For peer review only - http://bmjopen.bmj.com/site/about/guidelines.xhtml

The Nirmal district (erstwhile Adilabad district) has witnessed a few epidemiological studies over the years. The Andhra Pradesh Eye Disease Study (APEDS) was conducted in 1997-98, followed by Rapid Assessment of Cataract Surgical Services (RACSS), a couple of RAVI studies in 2014, and the current study in 2022 (Table 4). Among those aged ≥40 years, the prevalence of VI (<6/18 definition) was 35.2% in APEDS, which dropped to 13.7% in the 2014 RAVI study. The prevalence declined further to 9.7% in the current study. Among those aged 50 years and older, the prevalence of VI (<6/18 definition) was 50.7% in APEDS, which dropped to 21.6% in RACSS conducted in 2006-07, and remained stable in the 2014 RAVI study, declining to 16.2% in the current study. As different protocols have been used over the years, direct comparisons are limited by the different measurement methods. Nevertheless, VI is declining in this region, as indicated by the two recent RAVI studies using identical protocols.

Table 4: Prevalence of visual impairment in Nirmal district by reported various studies.

	**Year**	**Sample size**	**Moderate Visual Impairment** (Presenting visual acuity worse than 6/18 to 6/60)	**Severe Visual Impairment** (Presenting visual acuity worse than 6/60 to 3/60)	**Blindness** (Presenting visual acuity worse than 3/60)	**Total Visual Impairment** (Presenting visual acuity worse than 6/18)
		**n**	**%**	**%**	**%**	**%**
**≥40 years**						
APEDS*[19,20]	1997-98	840	28.5	1.8	5.0	35.2
RAVI †[14]	2014	2974	10.4	1.5	1.9	13.7
RAVI †	2022	2392	8.8	0.4	0.6	9.7
**≥50 years**						
APEDS *[19,20]	1997-98	521	40.3	2.9	7.5	50.7
RACSS ∫ [27]	2006-07	2160	13.6	4.8	3.2	21.6
RAVI † [14]	2014	1550	16.6	2.3	3.0	21.9
RAVI †	2022	1491	13.4	1.1	1.7	16.2

* *Andhra Pradesh Eye Disease Study.* †*Rapid Assessment of Visual Impairment.* ∫ *Rapid Assessment of Cataract Surgical Services.*

A good response rate, a randomly selected population-based sample, the use of an updated WHO definition of VI, and the inclusion of participants of wider age groups are the strengths of this study. After APEDS, this is the major study to report VI in the adult population in this region. However, a few studies have reported the VI in older age groups during this gap of

For peer review only - http://bmjopen.bmj.com/site/about/guidelines.xhtml

two decades. A higher proportion of women were examined in this study, which could be due to the migration of men to urban areas in search of work, a common occurrence in Telangana. The previous studies in this region also show a female preponderance.[11,14] Therefore, the overrepresentation of women could have overestimated the prevalence of VI in our study. Another inherent limitation of rapid assessment methods is the ascertainment of causes of VI. The major cause is considered based on the ease of remedy to address the VI. Often the prevalence of cataract and refractive errors are overestimated as they are easy to treat and correct respectively compared to posterior segment conditions. This limitation applies to the current study as well as the rapid assessment methodology was used.

In conclusion, a significant burden of VI is observed in the region. However, the declining trend in the prevalence of VI suggests that the eye care services in the region are improving. This study can be a guide for more focused efforts to address vision loss and achieve universal eye health in this region. Also, there is a need for integration and eye care services with primary health care as people with diabetes had a higher prevalence of VI. VI impedes the attainment of sustainable development goals and the overall quality of life. Efforts to address vision loss might have a ripple effect on the overall health and well-being of individuals, families, and communities, contributing towards sustainable development goals.

Acknowledgements: The authors thank the vision technicians (Sunil Anant Doke, Vaibhav Kumar Dave, Jalindar Kumar Doke, Degaon Ganesh, Gangadharolla Vamshi) and community eye health team (Yedla Sandeep, Akaram Chinnaiah, Laxman Sunkari, Gunde Rao Rahul, Ramnapoina Mahesh) for the assistance provided in data collection. The authors also thank Sandeep Dayal, Thirupathi Reddy Kumbham, Shekhar Konegari and Saptak Banerjee for monitoring the study. Abhinav Sekar is acknowledged for the language inputs on earlier versions of our manuscript. The authors thank the volunteers for their participation in the study.

Contributors: SM conceived the idea, designed and conducted the study, analyzed the data and wrote the manuscript. AC was involved in data collection and quality control. VKY, RSV, RCK, RN reviewed the earlier version of the manuscripts and provided the intellectual inputs.

Funding Support: This work was supported by the DBT Wellcome Trust India Alliance Clinical Research Centre Grant [grant number IA/CRC/19/1/610010] awarded to Dr Raja Narayanan and Hyderabad Eye Research Foundation, India.

For peer review only - http://bmjopen.bmj.com/site/about/guidelines.xhtml

Competing Interests: The authors report no conflicts of interest and have no proprietary interest in any of the materials mentioned in this article.

Data sharing statement: No additional data are available.

Ethics Approval: The study protocol was approved by the Institutional Review Board (IRB) of Hyderabad Eye Research Foundation, L V Prasad Eye Institute (LVPEI) (Reference ID: LEC-08173). This study was conducted in accordance with the tenets of the Declaration of Helsinki. Written informed consent was obtained from all the participants. For those aged less than 18 years of age, assent for the eye examination was obtained from the participant and written informed consent was obtained from the legal guardian.

REFERENCES

1. Bourne R, Steinmetz JD, Flaxman S, et al. Trends in prevalence of blindness and distance and near vision impairment over 30 years: an analysis for the Global Burden of Disease Study. The Lancet Global Health 2021;**9**(2):e130-e43 doi: https://doi.org/10.1016/S2214-109X(20)30425-3[published Online First: Epub Date]|.
2. Steinmetz JD, Bourne RRA, Briant PS, et al. Causes of blindness and vision impairment in 2020 and trends over 30 years, and prevalence of avoidable blindness in relation to VISION 2020: the Right to Sight: an analysis for the Global Burden of Disease Study. The Lancet Global Health 2021;**9**(2):e144-e60 doi: 10.1016/S2214-109X(20)30489-7[published Online First: Epub Date]|.
3. Marmamula S, Keeffe JE, Rao GN. Rapid assessment methods in eye care: An overview. Indian J Ophthalmol 2012;**60**(5):416-22 doi: IndianJOphthalmol_2012_60_5_416_100539 [pii] 10.4103/0301-4738.100539[published Online First: Epub Date]|.
4. McCormick I, Mactaggart I, Resnikoff S, et al. Eye health indicators for universal health coverage: results of a global expert prioritisation process. Br J Ophthalmol 2022;**106**(7):893-901 doi: 10.1136/bjophthalmol-2020-318481[published Online First: Epub Date]|.
5. Mactaggart I, Limburg H, Bastawrous A, Burton MJ, Kuper H. Rapid Assessment of Avoidable Blindness: looking back, looking forward. Br J Ophthalmol. 2019;**103**:1549-52
6. Marmamula S, Khanna RC, Narsaiah S, Shekhar K, Rao GN. Prevalence of spectacle use in Andhra Pradesh, India: Rapid Assessment of Visual Impairment (RAVI) project.

For peer review only - http://bmjopen.bmj.com/site/about/guidelines.xhtml

Clin Exp Ophthalmol 2013 doi: 10.1111/ceo.12160[published Online First: Epub Date]|.

7. Marmamula S, Madala SR, Rao GN. Rapid assessment of visual impairment (RAVI) in marine fishing communities in South India--study protocol and main findings. BMC Ophthalmol 2011;**11**:26 doi: 10.1186/1471-2415-11-26[published Online First: Epub Date]|.

8. Marmamula S, Madala SR, Rao GN. Prevalence of uncorrected refractive errors, presbyopia and spectacle coverage in marine fishing communities in South India: Rapid Assessment of Visual Impairment (RAVI) project. Ophthalmic Physiol Opt 2012;**32**(2):149-55 doi: 10.1111/j.1475-1313.2012.00893.x[published Online First: Epub Date]|.

9. Marmamula S, Narsaiah S, Shekhar K, Khanna RC. Visual Impairment among Weaving Communities in Prakasam District in South India. PLoS One 2013;**8**(2):e55924 doi: 10.1371/journal.pone.0055924[published Online First: Epub Date]|.

10. Marmamula S, Narsaiah S, Shekhar K, Khanna RC. Presbyopia, spectacles use and spectacle correction coverage for near vision among cloth weaving communities in Prakasam district in South India. Ophthalmic Physiol Opt 2013 doi: 10.1111/opo.12079[published Online First: Epub Date]|.

11. Marmamula S, Narsaiah S, Shekhar K, Khanna RC, Rao GN. Visual Impairment in the South Indian State of Andhra Pradesh: Andhra Pradesh - Rapid Assessment of Visual Impairment (AP-RAVI) Project. PLoS One 2013;**8**(7):e70120 doi: 10.1371/journal.pone.0070120[published Online First: Epub Date]|.

12. Marmamula S, Yelagondula VK. The feasibility of combining rapid assessment studies for measuring the progress toward Universal Eye Health. Indian J Ophthalmol 2023;**71**(5):2296-97 doi: 10.4103/IJO.IJO_2996_22[published Online First: Epub Date]|.

13. Marmamula S, Modepalli SB, Kumbham TR, Challa R, Keeffe JE. Prevalence of disabilities and non-communicable diseases in an elderly population in the Telangana state, India: a population-based cross-sectional study. BMJ open 2021;**11**(2):e041755 doi: 10.1136/bmjopen-2020-041755[published Online First: Epub Date]|.

14. Marmamula S, Khanna RC, Kunkunu E, Rao GN. Population-based assessment of prevalence and causes of visual impairment in the state of Telangana, India: a cross-sectional study using the Rapid Assessment of Visual Impairment (RAVI)

For peer review only

For peer review only - http://bmjopen.bmj.com/site/about/guidelines.xhtml

methodology. BMJ open 2016;**6**(12):e012617 doi: 10.1136/bmjopen-2016-012617[published Online First: Epub Date]|.

15. Marmamula S, Khanna RC, Narsaiah S, Shekhar K, Rao GN. Prevalence of spectacles use in Andhra Pradesh, India: rapid assessment of visual impairment project. Clin Exp Ophthalmol 2014;**42**(3):227-34 doi: 10.1111/ceo.12160[published Online First: Epub Date]|.
16. Marmamula S, Khanna RC, Shekhar K, Rao GN. A population-based cross-sectional study of barriers to uptake of eye care services in South India: the Rapid Assessment of Visual Impairment (RAVI) project. BMJ open 2014;**4**(6):e005125 doi: 10.1136/bmjopen-2014-005125[published Online First: Epub Date]|.
17. Marmamula S, Yelagondula VK, Khanna RC, et al. A Population-Based Cross-Sectional Study of Visual Impairment in West Godavari and Krishna Districts in Andhra Pradesh: Akividu Visual Impairment Study (AVIS). Ophthalmic Epidemiol 2022;**29**(4):411-16 doi: 10.1080/09286586.2021.1946829[published Online First: Epub Date]|.
18. Flaxman SR, Bourne RRA, Resnikoff S, et al. Global causes of blindness and distance vision impairment 1990-2020: a systematic review and meta-analysis. Lancet Glob Health 2017;**5**(12):e1221-e34 doi: 10.1016/S2214-109X(17)30393-5[published Online First: Epub Date]|.
19. Dandona L, Dandona R, Srinivas M, et al. Blindness in the Indian state of Andhra Pradesh. Invest Ophthalmol Vis Sci 2001;**42**(5):908-16
20. Dandona R, Dandona L, Srinivas M, et al. Moderate visual impairment in India: the Andhra Pradesh Eye Disease Study. Br J Ophthalmol 2002;**86**(4):373-7
21. Dandona L, Dandona R, Naduvilath TJ, et al. Burden of moderate visual impairment in an urban population in southern India. Ophthalmology 1999;**106**(3):497-504
22. Gupta N, Vashist P, Malhotra S, Senjam SS, Misra V, Bhardwaj A. Rapid assessment of visual impairment in urban population of Delhi, India. PLoS One 2015;**10**(4):e0124206 doi: 10.1371/journal.pone.0124206[published Online First: Epub Date]|.
23. Bhardwaj A, Vashist P, Singh Senjam S, Gupta V, Gupta N, Manna S. Rapid Assessment of Avoidable Visual Impairment in Two Coastal Districts of Eastern India for Determining Effective Coverage: A Cross-Sectional Study. J Ophthalmic Vis Res 2023;**18**(2):182-91 doi: 10.18502/jovr.v18i2.13185[published Online First: Epub Date]|.

For peer review only

For peer review only - http://bmjopen.bmj.com/site/about/guidelines.xhtml

24. Marmamula S, Yellapragada S, Khanna RC. Population Based Assessment of Prevalence and Causes of Vision Impairment in the North-eastern State of Tripura, India - The Tripura Eye Survey. Ophthalmic Epidemiol 2020;**27**(4):283-88 doi: 10.1080/09286586.2020.1731833[published Online First: Epub Date]|.

25. Senjam SS, Vashist P, Gupta N, et al. Prevalence of visual impairment due to uncorrected refractive error: Results from Delhi-Rapid Assessment of Visual Impairment Study. Indian J Ophthalmol 2016;**64**(5):387-90 doi: 10.4103/0301-4738.185614[published Online First: Epub Date]|.

26. Marmamula S, Khanna RC, Yellapragada S, Challa R, Mohd J, Rao GN. Temporal trends in the prevalence and causes of visual impairment in the South Indian state of Telangana: a population-based cross-sectional study. BMJ open 2019;**9**(7):e029114 doi: 10.1136/bmjopen-2019-029114[published Online First: Epub Date]|.

27. Khanna RC, Marmamula S, Krishnaiah S, Giridhar P, Chakrabarti S, Rao GN. Changing trends in the prevalence of blindness and visual impairment in a rural district of India: Systematic observations over a decade. Indian J Ophthalmol 2012;**60**(5):492-7 doi: IndianJOphthalmol_2012_60_5_492_100560 [pii]
10.4103/0301-4738.100560[published Online First: Epub Date]|.

28. Marmamula S, Keeffe JE, Narsaiah S, Khanna RC, Rao GN. Changing trends in the prevalence of visual impairment, uncorrected refractive errors and use of spectacles in Mahbubnagar district in South India. Indian J Ophthalmol 2013;**61**(12):755-8 doi: 10.4103/0301-4738.121149[published Online First: Epub Date]|.

For peer review only - http://bmjopen.bmj.com/site/about/guidelines.xhtml

Figure 1: Causes of visual impairment across the age groups.

For peer review only

For peer review only - http://bmjopen.bmj.com/site/about/guidelines.xhtml

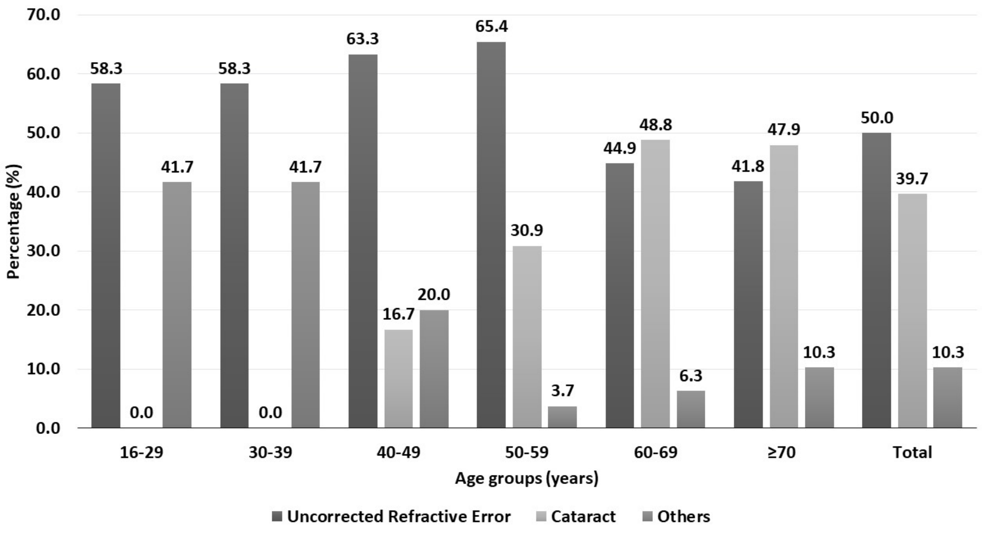

Causes of visual impairment across the age groups

338x190mm (96 x 96 DPI)

ID				

RAPID ASSESSMENT OF VISUAL IMPAIRMENT PROJECT – DATA COLLECTION FORM

Section A – Demographic Informatio

Address:

Name | **Age** | **Mobile**

Status
Circle the codes

1	Examined
2	Not available after 2 visits
3	Refused

Gender

1	Male
2	Female

Education Level

0	No education
1	Primary school (class 1-5)
2	High school (class 6-10)
3	Intermediate (class 11-12)
4	College (undergraduate)
5	Advanced studies (PG etc)
6	Others, specify

Occupation

0	Unemployed
1	Teacher / clerical jobs
2	Driver
3	Shop Keeper / Business
4	Labourer – Agriculture
5	Labourer – Other
6	Beedi rolling
7	Stopped working due to poor vision
8	Home duties only
9	Retired / stopped worked due to old age
10	Others, specify

Section B – Spectacle Information

Present Glasses

0	No glasses
1	Yes

Amount paid

Glasses - Type

0	No glasses
1	SV - Distance
2	SV- Near
3	Bifocals

Provider

0	No Glasses
1	LVP Vision Centre
2	LVP Service centre
3	Private Eye doctor / Clinic
4	Directly from the local optical shop
5	Govt. in a camp for no cost

Section C – Surgery Information and Systemic Conditions (Please enter the codes)

	RE	LE
**When was surgery done**		
**Costs of surgery**		

0	Not applicable
1	Free / Nonpaying
2	Paying **Amount**

	RE	LE
**Place of surgery**		

0	Not applicable
1	Eye camp
2	NGO hospital
3	Private hospital
4	Government hospital

Systemic Condition ***(Enter the code as appropriate)***

Condition	Duration (yrs)

Codes: 0= None, 1=HTN, 2= DM, 3= Heart disease, 4= Asthma, 5=Other, specify

For peer review only

Section D – Visual Acuity and Clinical Examination *(Enter the code as appropriate)*

**Visual acuity**	**RE**	**LE**	**BE**
Unaided - Distance			
Pinhole - Distance *(Only if <6/12)*			
Aided – Distance*			
Pinhole - Distance			
**PRESENTING VA (PVA)**			

Unaided – Near (Binocular)	
Aided - Near	
Add power used	+
Near vision with addition	

* Record aided vision with +10 for aphakia

(Codes: cf 3 mts=8; cf 2mts=9; cf 1m=10 PLPR=11; NOPL=12)

**Lens status**		**RE**	**LE**
1	Normal lens		
2	Obvious lens opacity/cataract		
3	Aphakia		
4	Pseudophakia		
5	No view of lens, why		

**Other Major Finding**			
0	None		
1	Corneal scar		
2	Pterygium		
3	Posterior capsular opacification		
4	Others		

Section E – Primary causes of Visual Impairment *(Enter the code as appropriate)*

Principal cause of presenting vision <6/12

		**RE**	**LE**	**BIN**
0	No visual impairment			
1	Refractive Error			
2	Uncorrected aphakia			
3	Cataract			
4	Surgery related complications			
5	Corneal opacity			
6	Phthisis or absent globe			
7	Glaucoma			
8	Posterior segment disorders			
9	Others, specify			

(BIN=least of the two)

Please Note:
Presenting VA= Aided VA if subjects has glasses;
Presenting VA= Unaided VA if subject has no glasses

Name of the examiner: ______________________

Signature/Date: ____________________________

For peer review only

STROBE 2007 (v4) Statement—Checklist of items that should be included in reports of *cross-sectional studies*

Section/Topic	Item #	Recommendation	Reported on page #
**Title and abstract**	1	(*a*) Indicate the study's design with a commonly used term in the title or the abstract	1
		(*b*) Provide in the abstract an informative and balanced summary of what was done and what was found	1
**Introduction**			
Background/rationale	2	Explain the scientific background and rationale for the investigation being reported	3
Objectives	3	State specific objectives, including any pre-specified hypotheses	3
**Methods**			
Study design	4	Present key elements of study design early in the paper	3
Setting	5	Describe the setting, locations, and relevant dates, including periods of recruitment, exposure, follow-up, and data collection	4
Participants	6	(*a*) Give the eligibility criteria, and the sources and methods of selection of participants	4
Variables	7	Clearly define all outcomes, exposures, predictors, potential confounders, and effect modifiers. Give diagnostic criteria, if applicable	4,5
Data sources/ measurement	8*	For each variable of interest, give sources of data and details of methods of assessment (measurement). Describe comparability of assessment methods if there is more than one group	4,5
Bias	9	Describe any efforts to address potential sources of bias	NA
Study size	10	Explain how the study size was arrived at	na
Quantitative variables	11	Explain how quantitative variables were handled in the analyses. If applicable, describe which groupings were chosen and why	6
Statistical methods	12	(*a*) Describe all statistical methods, including those used to control for confounding	6
		(*b*) Describe any methods used to examine subgroups and interactions	6
		(*c*) Explain how missing data were addressed	NA
		(*d*) If applicable, describe analytical methods taking account of sampling strategy	NA
		(*e*) Describe any sensitivity analyses	NA
**Results**			

For peer review only

Participants	13*	(a) Report numbers of individuals at each stage of study—eg numbers potentially eligible, examined for eligibility, confirmed eligible, included in the study, completing follow-up, and analysed	NA
		(b) Give reasons for non-participation at each stage	NA
		(c) Consider use of a flow diagram	NA
Descriptive data	14*	(a) Give characteristics of study participants (eg demographic, clinical, social) and information on exposures and potential confounders	7
		(b) Indicate number of participants with missing data for each variable of interest	NA
Outcome data	15*	Report numbers of outcome events or summary measures	7
Main results	16	(*a*) Give unadjusted estimates and, if applicable, confounder-adjusted estimates and their precision (eg, 95% confidence interval). Make clear which confounders were adjusted for and why they were included	7,8,9
		(*b*) Report category boundaries when continuous variables were categorized	6
		(*c*) If relevant, consider translating estimates of relative risk into absolute risk for a meaningful time period	6
Other analyses	17	Report other analyses done—eg analyses of subgroups and interactions, and sensitivity analyses	7,8,9
**Discussion**			
Key results	18	Summarise key results with reference to study objectives	10
Limitations	19	Discuss limitations of the study, taking into account sources of potential bias or imprecision. Discuss both direction and magnitude of any potential bias	12,13
Interpretation	20	Give a cautious overall interpretation of results considering objectives, limitations, multiplicity of analyses, results from similar studies, and other relevant evidence	12,13
Generalisability	21	Discuss the generalisability (external validity) of the study results	13
**Other information**			
Funding	22	Give the source of funding and the role of the funders for the present study and, if applicable, for the original study on which the present article is based	13

*Give information separately for cases and controls in case-control studies and, if applicable, for exposed and unexposed groups in cohort and cross-sectional studies.

Note: An Explanation and Elaboration article discusses each checklist item and gives methodological background and published examples of transparent reporting. The STROBE checklist is best used in conjunction with this article (freely available on the Web sites of PLoS Medicine at http://www.plosmedicine.org/, Annals of Internal Medicine at http://www.annals.org/, and Epidemiology at http://www.epidem.com/). Information on the STROBE Initiative is available at www.strobe-statement.org.

For peer review only
